# Maturation of Moristel in Different Vineyards: Amino Acid and Aroma Composition of *Mistelles* and Wines with Particular Emphasis in Strecker Aldehydes

**DOI:** 10.3390/foods11070958

**Published:** 2022-03-25

**Authors:** Ignacio Arias-Pérez, Ignacio Ontañón, Vicente Ferreira, Ana Escudero

**Affiliations:** Laboratory for Aroma Analysis and Enology, Instituto Agroalimentario de Aragón (IA2-Unizar-CITA), Department of Analytical Chemistry, Faculty of Sciences, Universidad de Zaragoza, 50009 Zaragoza, Spain; iarias@unizar.es (I.A.-P.); ionta@unizar.es (I.O.); vferre@unizar.es (V.F.)

**Keywords:** ripening, longevity, oxidation, fermentation, Ehrlich pathway, Strecker aldehydes, amino acids

## Abstract

The aim of this article was to assess the influence of the harvest date on the composition of amino acids and derived aromatic compounds in grape-*mistelle* and wine of the Moristel variety, in different vineyards. Two vineyards were sampled in 2016 and another one in 2017. At each sampling point, grapes were collected, destemmed, crushed and divided into four aliquots. The first three were fermented, and the latter was treated with ethanol, to produce 1-week macerates containing 15% ethanol (*v*/*v*)-*mistelles*. Overall, 10 *mistelles* and 33 wines were produced. Amino acids, Strecker aldehydes and aroma compounds were analysed. Amino acid profiles are characteristic of the vineyard and level of ripeness, converging with maturation. In fermentation, major amino acids, except proline, are consumed at a relatively fixed and specific tax, while consumption of 13 amino acids is determined by the ratios of alanine, glutamic acid, serine and threonine, with γ-aminobutyric acid. After fermentation, amino acid precursors to Strecker aldehydes are maxima in unripe and overripe samples, while Strecker aldehydes are maxima in unripe wines. No direct correlations between precursor amino acids in *mistelle* and aromatic compounds in wine have been found. Nevertheless, must amino acid profiles could determine wine aroma composition.

## 1. Introduction

It has been demonstrated that the amino acid composition of musts influences the aromatic composition of wine [1]. Amino acids are a highly important source of yeast assimilable nitrogen (YAN) during alcoholic fermentation and they are also precursors of some relevant aroma compounds during the same process (e.g., higher alcohols) [2,3,4,5]. For this reason, the nitrogen composition of the grapes, and particularly of the musts, will have notable consequences for the aromatic quality of the final wine [6,7,8]. 

The amino acid profiles of grapes depend on many factors, such as edaphoclimatic conditions [9,10], grape variety [11,12], viticultural practices [13], management of soil and of vegetation [14,15] and the level of maturation [16,17], among others. The maturation factor is especially interesting because different studies demonstrate not only the effects of grape ripeness on amino acid composition, but its decisive effect on the quality and aromatic profile of the wine [18,19]. This can be attributed to the fact that the synthesis of many of the aroma compounds and of their precursors occurs during ripening [20,21]. 

There is not an entirely clear understanding of the relationship between the aromatic compounds found in wine and the assimilation of amino acids by yeast during fermentation [14,22,23]. The amino acid metabolism of yeast is a complex process [3,24] that depends on many factors, such as the levels of amino acids and of ammonium, the strain of yeast and its specific metabolic requirements, or the balance of reduced and oxidized forms of the main cofactors (NADH/NADPH), among others [5,8,22,23,25]. Despite this complexity, previous studies relate the amino acid profile of the must to differences in the wine contents, in important aroma compounds of fermentative origin, such as branched acids and their esters, and higher alcohols and their acetates [1,5,23], all of them thought to be formed through the Ehrlich pathway. All these aroma compounds structurally derive from the amino acids, valine, leucine, isoleucine, methionine and phenylalanine. 

Strecker aldehydes (isobutyraldehyde, 2-methylbutanal, isovaleraldehyde, methional and phenylacetaldehyde) are also part of the Ehrlich route, although it is generally considered that they are mere intermediates, so that they do not accumulate significantly during fermentation. Strecker aldehydes can be also formed by oxidative degradation of the precursor amino acids [26,27,28], or even by the oxidation of their corresponding alcohols [28,29]. Strecker aldehydes, notably methional and phenylacetaldehyde, are the most important contributors to oxidation aroma notes of wines [29,30,31].

Strecker aldehydes can participate in many reactions with different reagents, such as SO_2_, polyphenols, amino acids, proteins and other species [32,33]. As the products of the reaction aldehyde-SO_2_ are reversible, it has been demonstrated that during wine oxidation, as wine SO_2_ is consumed, previously formed aldehydes can be released from their adducts [28,34], causing the deterioration of wine aroma. However, it is not yet clear whether the aldehydes were formed during fermentation or in some step of the long process of wine making [35]. The influence of grape maturation on the ability of wine to form these compounds is also unknown. 

Moristel is an old, red, grape variety (*Vitis vinifera*), traditionally used in the Somontano Designation of Origin in northern Aragón (Spain), which has been little studied [36]. It has a long vegetative cycle, produces wines with relatively low levels of alcohol and performs well in the face of drought and diseases [37], which could be a potentially promising variety for adapting to arid climates. Moristel wines and *mistelles*, obtained from grapes treated with ethanol, were examined out in this study. *Mistelles* are suitable matrixes for studying the aroma potential of grapes because they contain metabolites, potentially extracted during wine making, and are relatively microbiologically stable [35,38].

The present study aims to understand the influence of the harvest date on the amino acid and aromatic composition of grapes-*mistelles* and wines of the Moristel variety, focusing especially on Strecker aldehydes.

## 2. Materials and Methods

### 2.1. Reagents, Solvents and Standards

Dichloromethane (≥99.9%), ethanol (≥99.9%) and methanol (≥99.9%) for gas chromatography analyses were supplied by Fisher Scientific (Loughborough, UK). Methanol (≥99.9%) and acetonitrile (≥99.9%) of HPLC and LiChrolut EN resins were supplied by Merck (Darmstadt, Germany). Pure water was obtained from a Milli-Q purification system (Bedford, Germany). Semi-automated solid-phase extraction was carried out with a Vac Elut 20 station from Varian (Walnut Creek, CA, USA). Standards and reagents for aroma compounds and amino acids determination were supplied by Merck (Darmstadt, Germany), ChemService (West Chester, PA, USA), PolyScience (Niles, IL, USA), Lancaster (Eastgate, UK), Alfa Aesar (Karlsruhe, Germany), Panreac (Barcelona, Spain), Firmenich (Geneva, Switzerland), AromaLab (Planegg, Germany), Waters (Milford, MA, USA) and Oxford Chemicals (Hartlepool, UK). The purity of all these standards was above 96%.

### 2.2. Samples

The grape samples (cv. Moristel) came from three vineyards (A, B and C) harvested in different vintages: 2016 (vineyards A and B) and 2017 (vineyard C). The characteristics which define the selected vineyards are summarized in Table S1 of Supplementary Material. For the study of maturation development, a maximum of four harvest points were selected, with approximately one week’s separation between each (42, 49, 56 and 62 days *postvéraison*). Furthermore, the winery usually harvests 49 days *postvéraison*. The fruits were collected at each harvest date following a random design within each block (A and B in 2016, and C in 2017). The grapes were harvested manually in 15 kg boxes. The gathering of samples was adapted to the geography of the vineyard and the technical possibilities offered by the winery. Whenever possible, we avoided gathering grapes from the borderlines of the vineyard. The samples are shown in Table 1.

#### 2.2.1. Grape Samples and Ethanolic Musts (*Mistelles*)

For the study of the grapes, 10 *mistelles* were elaborated. These are beverages prepared from grape must to which a quantity of alcohol is added to impede fermentation. One *mistelle* was elaborated for each vineyard and maturation point (except first point of vineyard C).

Fifteen kilograms of grapes were first destemmed and crushed in the presence of 15% (*p*/*p*) ethanol (≥99.9%) and 5 g hL^−1^ of potassium metabisulfite. After 7 days’ macerating at 15 °C, the *mistelles* were pressed, filtered (obtaining an approximate total volume of 11 L) and stored at 5 °C in the dark.

Analysis of free aromas and amino acids was carried out eight months after elaboration. The samples are shown in Table 1.

#### 2.2.2. Wine Samples

Thirty-three samples of Moristel wine were elaborated in the same conditions as those described by Arias-Pérez et al. [19]. The micro-vinifications were carried out in the Pirineos Winery in D.O. Somontano and followed the same fermentation protocol. All fermentations were carried out in triplicate and these wines are described in Table 1.

For processing the wines, we carried out fermentation on 70-litre steel tanks, in which 40 kg of grapes were vinified. The grape of each vineyard and maturation point was processed (destemmed/crushed) independently and in triplicate. Then the paste was treated with SO_2_ (50 mg L^−1^), and a dose of 0.8 mL hL^−1^ of pectolytic enzymes (Endozym ICS 10 Rouge, AEB, Stuttgart, Germany) was added. They were inoculated with *Saccharomyces cerevisiae* (30 g hL^−1^ of Lalvin ICVD-254 (Lallemand, Montreal, Canada)). During the entire fermentation, density and temperature were monitored, with measurements taken twice daily. This process lasted between 11 and 14 days. Once alcoholic fermentation was finished, the wines were inoculated with *Oenococcus oeni* (10 mg L^−1^), strain Lalvin VP41 (Lallemand), and the concentration of malic acid was controlled. Once malolactic fermentation was completed (30–37 days), the wine’s total SO_2_ was corrected to 50 mg L^−1^ it was filtered, bottled with natural cork closures and stored in the dark at 20 °C. 

Aromas and amino acids were analysed eight months after the samples described in Table 1 were bottled.

### 2.3. Methods

#### 2.3.1. Oenological Parameters

The progress of fermentations was monitored by daily measurement of the density and temperature of the must wine. Fermented wines were further analysed to assess pH, total acidity (TA), volatile acidity (VA), total polyphenol index (TPI-I280), colour index (CI), alcoholic degree, reductive sugars and levels of malic and lactic acids. In the case of *mistelles*, only pH and TPI were assessed. All these measurements were carried out in accordance with the methodology established by the Office International de la Vigne et du Vin [39]. Results are given in Tables S2–S4 of the Supplementary Material.

#### 2.3.2. Quantitative Analysis of Major Aroma Compounds 

The determination of major aroma compounds in 10 *mistelles* and 33 wines was carried out using a variation of the method described by Ortega et al. [40]. This consists of liquid–liquid micro-extraction with dichloromethane and analysis in a gas chromatograph with a flame ionization detector (GC-FID). Areas of analytes normalized to one of the selected internal standards (4-hydroxy-4-methyl-pentanone, 2-butanol, 4-methyl-2-pentanol, 2-octanol, heptanoic acid and ethyl heptanoate), were further interpolated in the calibration graphs built with synthetic wine models (4 g L^−1^ tartaric acid, 13% ethanol, 10 g L^−1^ glycerin, 7 mg L^−1^ quinine, 75 mg L^−1^ arabic gum, 100 mg L^−1^ tannic acid, 25 mg L^−1^ tannin and pH 3.5). 

#### 2.3.3. Quantitative Analysis of Minor and Trace Aroma Compounds

Minor aroma compounds were extracted by solid-phase extraction (SPE) and were further analysed by gas chromatography coupled to an ion trap mass spectrometry detection system (GC–MS), as reported by López et al. [41]. Ion peak areas using the most selective and quantitative m/z (mass to charge ratio) ratios were normalized by the area of one of the internal standards (2-octanol, 3,4-dimethylphenol and 3-octanone) and transformed into concentration via interpolation in the calibration plots built via the analysis of synthetic wines (same composition as 2.3.2) with known amounts of analytes. 

#### 2.3.4. Determination of Total Strecker Aldehydes

The various odour-active Strecker aldehydes (isobutyraldehyde, 2-methylbutanal, isovaleraldehyde, methional, phenylacetaldehyde) in their total forms (free plus bound) were determined by headspace-SPME-GC-MS after breaking complexes with glyoxal. The method is described by Bueno et al. [42]. 

#### 2.3.5. Quantitative Analysis of Amino Acids

Amino acids—alanine (ALA), asparagine (ASN), arginine (ARG), aspartic acid (ASP), cysteine (CYS), γ-aminobutyric acid (GABA), glutamine (GLN), glutamic acid (GLU), glycine (GLY), histidine (HIS), isoleucine (ILE), leucine (LEU), lysine (LYS), methionine (MET), ornithine (ORN), phenylalanine (PHE), proline (PRO), serine (SER), threonine (THR), tyrosine (TYR) and valine (VAL)—ammonium and internal standard (α-aminobutyric acid) were treated by a derivatization strategy with 6-aminoquinolyl-N-hydroxysuccinimidyl carbamate (AQC) and were then determined by HPLC with a fluorescence detector according to the method proposed by Hernández-Orte et al. [43]. Peak areas, normalized by those of the internal standard were interpolated in the calibration plots built via the analysis of synthetic solutions containing known amounts of amino acids. 

### 2.4. Data Analysis

Analytical results were processed by one-factor analysis of variance (ANOVA) with maturity and vineyard as factors. One-factor ANOVA was further used to evaluate the effects of maturity for each vineyard. For significant effects, a Fisher post-hoc pairwise comparison (95%) test was performed.

In order to have an overview of all the samples, a principal component analysis (PCA) was carried out with *mistelle* amino acid concentrations (absolute and relative data) and wine amino acid concentration (relative contents) in the samples of three different vineyards. All analyses were carried out with XLSTAT (2018 version).

Correlation studies, linear least-squares regression and simple Student *t* tests were directly carried out with Excel 2016 (Microsoft, Washington, DC, USA).

## 3. Results and Discussion

Vineyards A and C had good physiological, phytosanitary condition and vegetative equilibrium, while B was unbalanced vineyard with an excess of vigour. These differences become obvious when comparing the ratio of exposed leaf area (ELA)/yield, a parameter that relates the vineyard exposed leaf area with its yield. Values of this ratio between 0.8 and 1.2 m^2^ kg^−1^ are considered optimal [44] reflecting an appropriate balance between vegetative growth and yield. Vineyards A and C gave ELA/yield values of around 1 m^2^ kg^−1^, while these values for vineyard B was around 0.6 m^2^ kg^−1^. Plot B had irregular vegetative development, and clusters of these vineyards showed low compactness and small berries, while others were compact with much larger grapes, which led to phytosanitary problems. The vineyards characteristics are shown in Table S1 of the Supplementary Material.

In this work, grape samples were crushed and macerated in the presence of alcohol and then pressed to obtain *mistelles* in order to ensure a sufficient extraction of polyphenolic material, similar to that observed during alcoholic fermentation and obtaining stable samples. Ten different *mistelles* were obtained from the three different vineyards, two sampled in 2016 and one in 2017. Thirty different wines, excluding three samples whose fermentations were problematic (BT4.1 and BT4.3, which showed elevated volatile acidity, and AT3.1, which showed an abnormal state of oxidation), were finally considered in the study. The day when the grapes were harvested and processed, must samples were analysed with the basic oenological parameters in the winery (Table S2 of the Supplementary Material). *Mistelles* and wines were characterised by classic oenological parameters, and were further analysed by GC-FID, GC-MS and HPLC for determining volatile compounds and amino acids. The complete sets of results can be found in Tables S3 and S4 of the Supplementary Material.

### 3.1. Chemical Changes in Grapes during Maturation

#### 3.1.1. Amino Acid Profiles

**Figure 1:**
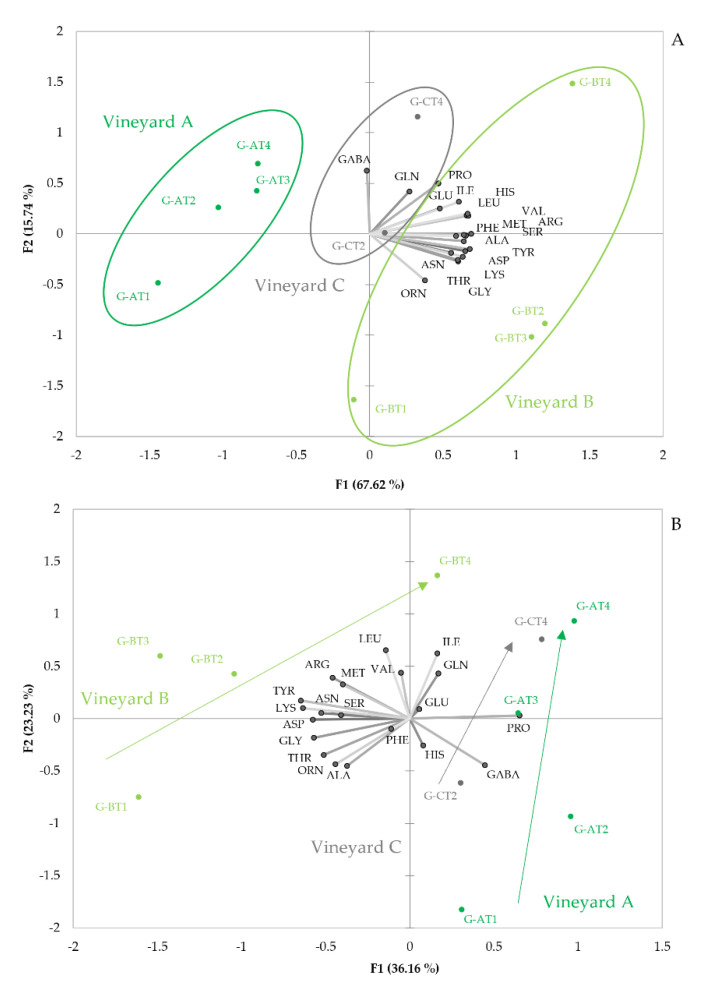
Amino acid variability with maturation in the three different vineyards sampled in the study. The plots give the projections of the sample scores and variable loadings in the planes formed by the two first Principal Components obtained in the PCA study carried out with amino acid data from 10 different *mistelles* made with representative grape samples from different ripeness and three different vineyards. (**A**), absolute data; (**B**), relative data (amino acid concentration in %).

Figure 1A shows the PCA plot obtained from amino acid data from the *mistelles*. As can be seen, samples are distributed in the PCA plane, attending first, to the vineyard they come from and, second, to the degree of ripeness. Samples from each vineyard are grouped forming right-leaned ellipses, distributed along the first component. The rotated axis, providing maximum difference between vineyards, follows closely the direction marked by the variable loading of ORN. It should be observed that all amino acids, except GABA (R = −0.44), PRO (R = −0.05) and GLN (R = −0.38), are positively correlated to such vector. This implies that the primary difference between samples from the different vineyards is the absolute content in amino acids, other than PRO and GLN and, particularly, the ratios between ORN and GABA or between the summation of all the amino acids most correlated to ORN and GABA. Samples from vineyard A contain the lowest amounts of amino acids, while those from vineyard B contain maxima levels and those from vineyard C have intermediate levels. The ratios ORN/GABA or LYS + THR + ORN + GLY + ASN + TYR + PHE/GABA take significantly different values for each vineyard and could be used for classification. The higher levels of amino acids in grapes from vineyard B are consistent with the fact that vines in this plot were more vigorous. More vigorous plants have higher photosynthetic activity and, hence, higher protein synthesis, hence, higher amino acid levels [14]. 

Figure 1A also reveals that grape maturity increases, following a diagonal from left-down to right-up of the plot. It is most remarkable that all amino acids, except ornithine, are positively linked to this diagonal line, representing ripeness, which suggests that during maturation, there is, essentially, an accumulation of amino acids, accompanied by some changes in amino acid profiles. These changes consist mainly of the relative enrichment of PRO, ILE, LEU, VAL and HIS. The general amino acid increase with maturity has been previously reported in some grape varieties [4,10,16,45]. The relevance of proline as a maturation driver is also known, since this amino acid is synthesized to counterbalance hydric stress [2,46], which explains its higher levels in over-matured and raisinized grapes. It is also worth mentioning, that this amino acid cannot be consumed by yeast under fermentation conditions [47], because its metabolism occurs in aerobiosis [16]. 

Changes in the amino acid profiles associated to maturation are best seen in the plot given in Figure 1B. This representation corresponds to the PCA carried out with amino acid data, normalized by the total amount of amino acids in each sample. The plot clearly shows that unripe grapes from the different vineyards are most dissimilar and that, during maturation, there is a convergence towards a common amino acid profile, located in the right-upper part of the plot; i.e., matured samples are characterized by positive scores of PC1 and positive scores of PC2. Different vineyards differ mainly in the relative proportions of GABA and PRO, on the one hand, and of the rest of amino acids (excluding ILE, GLN, GLU and HIS), on the other hand. Low-vigorous vineyards A and C are characterized by the highest proportions of proline and GABA, while high-vigorous vineyard B has minima proportions of these two amino acids. During maturation, however, differences in the relative content of those two amino acids shrink, so that matured samples acquire a common amino acid profile, characterized by relatively high proportions of PRO, GLN, ILE, VAL and LEU and relatively low proportions of ALA, THR, ORN, GLY, ASP, LYS and TYR. The large differences between unripe samples suggest the existence of some vineyard-specific changes during maturation. As can be seen, in vineyard A, there is an enrichment in GLN, ILE, VAL and LEU, while in vineyard B, there is a neat enrichment in PRO and GLN, but levels of ILE, VAL and LEU, remain unaltered. Nevertheless, some general maturation indices could be defined by the following ratio:

$$\frac{\mathrm{PRO}+\mathrm{GLN}+\mathrm{ILE}+\mathrm{VAL}+\mathrm{LEU}}{\mathrm{ORN}+\mathrm{LYS}+\mathrm{GLY}+\mathrm{THR}}$$

or by:

$$\frac{\mathrm{PRO}}{\mathrm{ORN}}$$

These are significantly correlated with traditionally used indices, such as the Cillis and Odifreddi index (concentration of sugars/total acidity) and the Goded index (must density/total acidity). Correlation coefficients are 0.71 (*p* = 0.01) and 0.72 (*p* = 0.01) for the Cillis and Odifreddi index and 0.65 (*p* = 0.03) and 0.67 (*p* = 0.02) for the Goded index, for the two quotients, respectively. 

#### 3.1.2. Volatile Fraction

The volatile composition of *mistelles* is also related to vineyard and maturation level, although the weight of these factors is not as dominant as it was in the case of amino acids.

**Figure 2:**
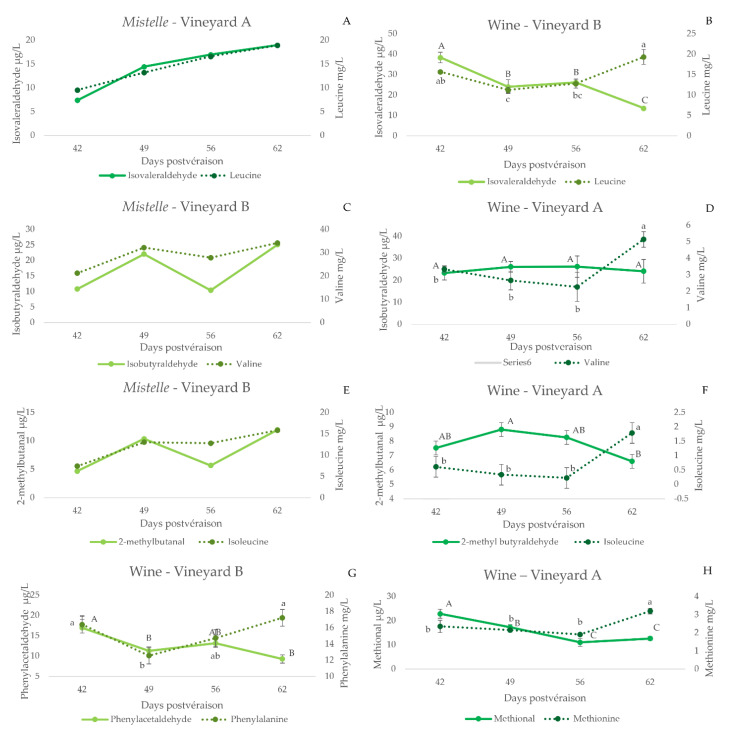
Evolution of some Strecker aldehydes and their amino acid precursors in *mistelle* samples (**A**,**C**,**E**) or in wines (**B**,**D**,**F**,**G,H**) made with grapes sampled from specific vineyards at different degrees of ripeness (expressed as days post-veraison). Error bars are calculated as s/(*n*)^1/2^(s, standard deviation; *n* = 3). Different letters indicate significant differences in the content of the compounds (*p* < 0.05) (Fisher post hoc test) (upper case: aldehydes; lower case: amino acids).

The evolution of Strecker aldehydes in *mistelles* is shown in Figure 2A,C,E. Strecker aldehydes increase with ripeness, except phenylacetaldehyde. In general, and as can be seen in Figure 2A,C,E, the increases in precursor amino acid parallel those of the corresponding aldehyde. This is corroborated by the significant positive correlations between the precursor amino acid and the Strecker aldehyde (leucine-isovaleraldehyde, R = 0.99, *p* < 0.01; valine-isobutyraldehyde, R = 0.81, *p* < 0.01; isoleucine-2-methylbutanal, R = 0.78, *p* < 0.01; and methionine-methional, R = 0.79, *p* = 0.01), except for phenylacetaldehyde (phenylalanine-phenylacetaldehyde, R = 0.40 *p* = 0.29). To the best of our knowledge, there are no previous reports indicating the presence of Strecker aldehydes in healthy musts. Our experimental approach, however, cannot determine whether these compounds are already present in the grape, or are formed by Strecker degradation during the production of the *mistelle*. Should this be the case, they could be formed by the reaction of the precursor amino acid with a quinone [26,27,28]. The poor formation of phenylacetaldehyde could be related to an already suggested smaller reactivity of phenylalanine to polyphenols [48], or to the inhibition of the reaction by the presence of glucose [30].

Regarding varietal aroma compounds, it is known that grapes contain very small amounts of free aroma compounds, which are slowly formed from precursors. The amounts reported here refer to the aroma compounds present in free form, after eight months of bottle conservation at the specific pH and sugar level of each *mistelle*. The few remarkable observations are summarized in Figure S1 of the Supplementary Material. TDN (1,1,6-trimethyl-1,2-dihydronapthalene) levels seem to decrease with maturation, except in the last sampling point of Vineyard A. Similar patterns are observed for vistispiranes, Riesling acetal and for γ-nonalactone (Table S3 of the Supplementary Material). These peculiar evolutions, however, seem to be closely related to the pH of the *mistelles* (see Figure S1 of the Supplementary Material). It is apparent that higher levels of TDN and γ-nonalactone are related to lower pHs. This, together with opposite evolutions with maturity, reported by other authors both for TDN [21] and γ-nonalactone [49], suggests that the observed content is more related to the higher rates at acidic pHs of the different reactions responsible for the formation of TDN and the other related norisoprenoids and γ-nonalactone, than to the real existence of higher levels of specific precursors of these molecules. This fact is consistent with the significant negative correlations between levels of these aroma compounds and pH (TDN, R = −0.60, *p* = 0.05; vitispirane A, R = −0.66, *p* < 0.05; vitispirane B, R = −0.65, *p* < 0.05; γ-nonalactone, R = −0.70, *p* < 0.05). The levels of some other aroma compounds in the *mistelles* also bear negative correlations with pH, such as α-terpineol (R = −0.61, *p* = 0.05) and ethyl isovalerate (R = −0.73, *p* < 0.05). All these compounds are also formed through pH-dependent chemical reactions; α-terpineol is one of the products in which linalool and geraniol decompose, while the esters are formed quicker in acidic media. Finally, levels of ethyl vanillate tend to increase with maturation (Table S3 of the Supplementary Material), in accordance with previous reports [50].

### 3.2. Wines and Their Relationship to Original Mistelles

#### 3.2.1. Amino Acid Changes during Fermentation

The *mistelles* from Moristel are particularly rich in proline (0.5 to 1.2 g L^−1^), followed by THR, ARG, GABA and ALA, all with average contents between 0.1 g L^−1^ and 0.5 g L^−1^. Proline and arginine were present in the highest concentrations in must, which is true of most varieties of red grapes [4,16,17,45]. Proline can be found in a wide range of concentrations in must, from 0.008 to 0.82 mg L^−1^ [4]. For example, Hernández-Orte et al. [16] found proline concentrations from 0.03 to 0.49 g L^−1^ in the Tempranillo variety, evaluated in 3 years in the Somontano region.

The maxima variability is observed for ARG levels (RSD (%) = 62%). Contents in GLU, SER, HIS and ASP, are also above 30 mg L^−1^, while average levels of GLN, ILE, ASN, GLY and MET were below 12 mg L^−1^. Levels of VAL, LEU and PHE, the amino acids together with ILE and MET precursors of Strecker aldehydes, were 24, 21 and 18 mg L^−1^, respectively. Data are shown in the Supplementary Material.

The wines contained smaller levels of nearly all amino acids, except PRO, which as previously mentioned, is not consumed by yeast. Only THR, ALA and GABA have average levels between 25 and 45 mg L^−1^. ARG is nearly completely consumed, so that its average levels in wine are close to 14 mg L^−1^. Levels of LYS, ASN and GLY in wines were slightly above those measured in *mistelles*. It is not clear whether this is the consequence of instrumental imprecision (concentrations are very low) or whether these amino acids are really synthesized and excreted by yeasts during fermentation. 

In relative terms, the amino acids most consumed are ARG (93%), followed by THR (89%), GABA and SER (85–81%) and HIS and ALA (80–75%). Leaving aside the four amino acids not consumed during fermentation (ASN, GLY, PRO, LYS), the amounts consumed are significantly correlated to the initial level of amino acid, in all cases, except those of TYR and PHE. The correlation coefficients were so high for highly consumed amino acids (0.999 ARG, 0.984 SER, 0.982 THR, 0.966 GLU, 0.954 ALA, 0.944 GABA, 0.890 HIS, 0.871 VAL and 0.817 ASP; as shown in Table 2) that for these major amino acids, the amount consumed by yeast is strictly proportional to the available amino acid in the must, suggesting that yeasts use a relatively constant fraction of each available and easy-to-adsorb amino acid. In this process, it must be taken into consideration that some amino acids are unstable in must and wine and a large variation in nitrogen content takes place during the alcoholic fermentation [4,6,24].

The remaining amounts of amino acids in the wine are also significantly correlated with initial amino acid concentration, in many instances. Correlations are high only in the non-assimilated amino acids (ASN, TYR and to a lesser extent LYS), are non-significant in some of the most consumed (HIS and ARG) and in the cases of ASP, MET and ORN, as shown in Table 2. Regarding the amino acids significantly consumed during fermentation, many of their levels in wine seem to be highly determined by the absolute levels of the most concentrated assimilable amino acids of the must (THR, ARG, ALA, GABA, GLU and SER), and particularly to the ratios of some of them with GABA. In fact, as the amino acids in wine are positively correlated with the content of the same amino acid in *mistelle*, some of these correlations improved with the same Aa/GABA ratio, especially with HIS, GLU, MET, ALA, VAL or LEU. These results are summarized in Table 3, where it can be seen that the levels of 13 amino acids of wine can be satisfactorily modelled (≥75% variance explained), with those simple correlations. In three other cases (ASP, ALA and SER), the ratios were significant but the variance was between 0.70 and 0.74.

In all cases, the relationship between amino acid level and the ratio is positive, which suggests that the quotients between must main amino acids (particularly ALA, GLU, SER and eventually THR) and GABA are likely determinant for the transport of the amino acid to the interior of the yeast cell. Transport and, therefore, (apparent) consumption seem to be favoured by small levels of the “principal amino acid” (where principal refers to ALA, GLU, SER or THR) and high levels of GABA (small ratios principal aa/GABA), so that remaining levels of amino acid in the wine are low. On the contrary, high levels of ALA, GLU and SER and small levels of GABA (high ratios principal aa/GABA) seem to be difficult in the transport and, hence, the consumption, of the amino acid.

This hypothesis should be supported by the existence of clear correlations between the amounts of amino acid apparently consumed and the quotients principal aa/GABA (data not shown). Those direct correlations exist but do not reach significance, because of the higher uncertainty of the measurement of consumptions (being a difference, require double measurement) and particularly because of the strong correlations between consumptions and original amounts (Table 2). To overcome this, the proportion of amino acid consumed should be modelled. In this case, there are strong and significant negative correlations between these proportions and the quotients 1/GABA, ALA/GABA, GLU/GABA, supporting the validity of the previous hypothesis (Table S5 of the Supplementary Material).

#### 3.2.2. Amino Acid Consumption and Wine Volatiles

A surprising result is that either *mistelle* contents or levels of metabolised Strecker-precursor amino acids (VAL, LEU, ILE, MET and PHE) are not related at all to levels of the major by-products of their consumption, which are higher alcohols, as shown in Table 2. This suggests that the non-polar amino acid precursors of higher alcohols are not primarily used by yeast as sources of N through the Ehrlich pathway. These amino acids bear, however, some relevant correlation with some other specific aroma molecules formed through the Ehrlich pathway, such as ethyl isobutyrate and ethyl isovalerate. These fruity esters are structurally related to valine and leucine, respectively, and their levels in wine are significantly and negatively correlated to levels of these two amino acids in *mistelles* (R = −0.78 ethyl isobutyrate vs. valine, significant at *p* < 0.01; R = −0.72 ethyl isovalerate vs. leucine, significant at *p* < 0.05). Levels of ethyl isovalerate are additionally correlated to the consumed amounts of leucine (R = −0.83, *p* < 0.01). The fact that levels of isobutyric and isovaleric acids are, however, not clearly related to these amino acids, reveals the existence of quite complex regulations of these metabolisms. These results support observations from Crépin et al. [24], arguing that catabolism of consumed amino acids plays a less important role in the formation of volatile compounds and that precursors of keto acids, required for synthesis de novo, have their source principally in catabolism of sugars. 

However, data also suggest that the overall composition of amino acids of the *mistelles* and the profile of amino acid consumption during fermentation are key determinants of the levels of aroma compounds, formed through the Ehrlich route, confirming early works, demonstrating the relationships between must amino acid profiles and wine aroma [1], as can be seen in Table 2. This can be seen, for instance, in the strong correlations between methionol, β-phenylethanol and isovaleric acid with glutamic acid. Levels of these aroma compounds are strongly and negatively correlated to the consumed amounts of that amino acid (R = −0.94, −0.93, and −0.92, all significant at *p* < 0.001, respectively). Further, the correlation between isobutyl acetate and levels of ornithine consumed is also worth mentioning (R = −0.92, *p* = 0.006). Other amino acids, showing a strong influence on wine aroma composition, including Strecker aldehydes, are ASN, ALA, MET, ILE, LEU and LIS (See Table 2 (mw)). 

Not surprisingly, given the strong relationships between the *mistelle* and the wine amino acid compositions, there are also strong significant correlations between the residual amino acid content of wine and its aromatic profile (data not shown). 

#### 3.2.3. Amino Acid Profiles in Wines

**Figure 3:**
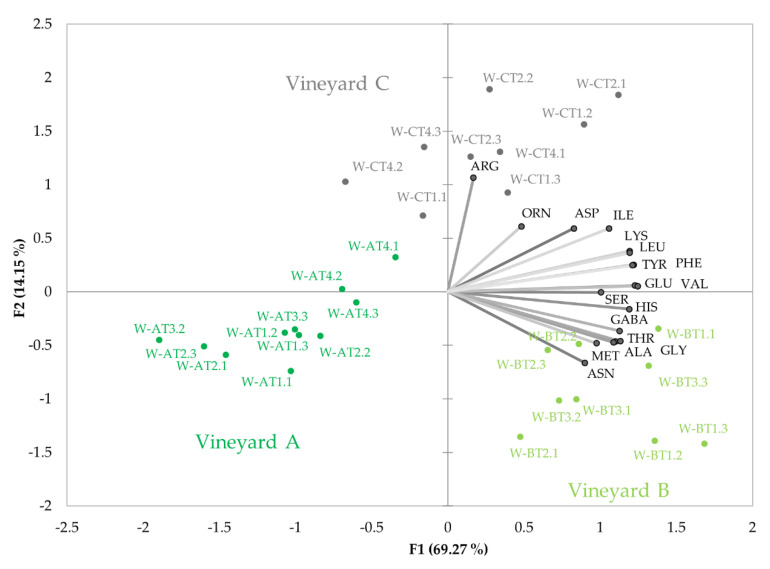
The plot shows the variability with maturation of residual wine amino acid relative contents (%) in the 30 wines made with grapes harvested from the three vineyards sampled in the study. The plot gives the projections of the sample scores and variable loadings in the planes formed by the two first Principal Components obtained in the PCA study carried out with amino acid data excluding proline.

As previously mentioned, the high correlations between wine and *mistelle* amino acids mean that the influence of maturation and vineyard on must amino acid composition also translates to wine. One of the consequences is that wines from vineyards with greater vigour (vineyard B) have higher levels of remaining amino acids. The effects of vineyard on wine residual amino acid profiles, excluding PRO, can be seen in Figure 3. The plot reveals a quite different amino acid profile to that observed in unfermented samples. As can be seen, each vineyard seems to have a more specific amino acid profile, so that the common profile acquired by matured samples, shown in Figure 1B, becomes quite distorted by fermentation. 

In this case, however, maturity is not clearly identified in the plot. This is, in part, because PRO has been excluded, but also because fermentation has cancelled some of the strong effects of maturity observed in must amino acid contents.

#### 3.2.4. Strecker Aldehydes

**Figure 4:**
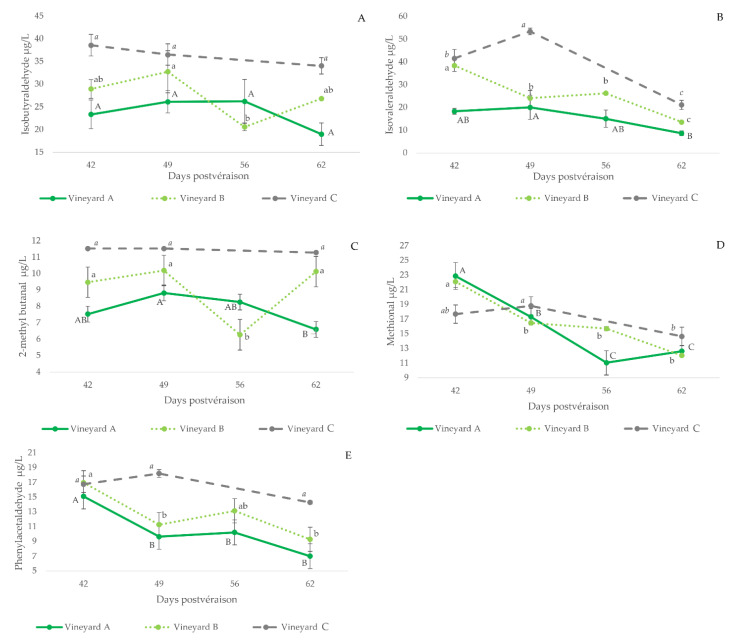
Evolution of the levels of Strecker aldehydes (µg/L) measured in wines made with grapes harvested at different degrees of ripeness in the three different studied vineyards. Error bars are calculated as s/(*n*)^1/2^(s, standard deviation; *n* = 3). The letters indicate significant differences in aldehyde content ((*p* < 0.05) (upper case: vineyard A; lower case: vineyard B; italics: vineyard C). (**A**): isobutyraldehyde; (**B**): isovaleraldehyde, (**C**): 2-methyl butanal; (**D**): methional; (**E**): phenylacetaldehyde.

The contents of Strecker aldehydes in wines made with grapes of different maturation states are shown in Figure 2 (plots B, D, F, G and H), together with those of the precursor amino acid, while the plots in Figure 4 compile the evolutions of the aldehydes for all the vineyards sampled. As can be seen, wines made with unripe grapes have higher levels of Strecker aldehydes. This is particularly true for isovaleraldehyde (3B), methional (3D) and phenylacetaldehyde (3E). These results are consistent with the oxidized aroma nuances noted in the wines made with these unripe grapes [36].

These higher levels of Strecker aldehydes in wines made with unripe grapes are likely related to the higher levels of the precursor amino acids in wine (leucine-isovaleraldehyde, R = 0.55, *p* = 0.001; valine-isobutyraldehyde, R = 0.42, *p* = 0.02; isoleucine-2-methylbutanal, R = 0.49, *p* = 0.005; methionine-methional, R = 0.42, *p* = 0.02 and phenylalanine-phenylacetaldehyde, R = 0.56, *p* < 0.001), to the smaller polyphenol content of wines made with unripe grapes and to the observed higher difficulties of yeasts to renew reduced forms of NADH and NADPH cofactors in musts from unripe grapes [19]. 

Higher polyphenol levels would limit levels of aldehydes by the formation of vitisin-type structures and ethyl-bridge-type condensed forms. In fact, TPI bears a significant negative correlation with Strecker aldehydes, except methional (isovaleraldehyde, R = −0.74, *p* < 0.001; isobutyraldehyde, R = −0.73, *p* < 0.001; 2-methylbutanal, R = −0.62, *p* < 0.001; methional, R = −0.13, *p* = 0.49; and phenylacetaldehyde, R = −0.67, *p* < 0.001). 

It should be noted, however, that wines made with later harvested grapes also contain higher levels of the amino acid precursors of Strecker aldehydes, as shown in Figure 2, which is a likely consequence of the higher levels of amino acids available in those musts. The practical consequence is that from the points of view of wine quality and longevity, harvest date has to take place in the period in which neither amino acid precursors or Strecker aldehydes are maxima.

## 4. Conclusions

The amino acid content of *mistelles* of Moristel is characteristic of the vineyard of origin and level of ripeness. The ratios ORN/GABA or LYS + THR + ORN + GLY + ASN + TYR + PHE/GABA take specific values for each vineyard. 

During maturation, all amino acids accumulate, but at different rates, so that *mistelles* become enriched in PRO, ILE, LEU, VAL and GLN and relatively impoverished in ORN, LYS, GLY and THR. In relative terms, the amino acid profiles of unripe samples from different vineyards show strong differences in the relative contents of GABA and PRO. These differences shrink with maturation, so that matured samples show relatively similar amino acid profiles. 

Strecker aldehydes and their precursor amino acids were found at higher levels in *mistelle* samples made with more matured grapes.

Leaving aside PRO, main must amino acids are largely consumed by yeasts, at relatively fixed and amino acid-specific taxes, so that levels consumed were proportional to the original content. The consumption of minor amino acids is, however, strongly and negatively dependent on the ratios between ALA, GLU, SER and, eventually, THR with GABA, so that the levels of 13 amino acids can be well predicted by those ratios. 

In spite of the deep modification of amino acid profile during fermentation, wines from different vineyards keep different amino acid profiles. However, the influence of maturation is much reduced.

While there is no obvious direct link between the must content in the amino acids involved in the Ehrlich pathway and the wine content in aromas produced through it, there is clear evidence that the amino acid profile of must strongly determines aroma formation. Amino acids with more influence are GLU, ORN, ASN, ALA, MET, ILE, LEU and LIS.

Early harvested grapes produced wines with more Strecker aldehydes and more amino acid precursors. Both decrease with maturation until the final maturation point, in which the amino acid content increases, which suggests that there is an optimal maturation point, with low levels of Strecker aldehydes and minima levels of their amino acid precursors.

## Figures and Tables

**Table 1:** Samples Table. Micro-vinifications and *mistelles* made with Moristel grapes with different degrees of maturation from plots with different features. The first letter of each abbreviation refers to the samples (G = *mistelles* and W = wine). The second letter is the vineyard (A, B or C); Tx is the harvest time (T1: 41–42 days *postvéraison*, T2: 48–49 days *postvéraison*, T3: 56 days *postvéraison* and T4: 60–63 days *postvéraison*). The last number is the sample (1, 2 or 3).

Year 2016				Year 2017			
Data	Vineyard	*Mistelle*	Wine	Data	Vineyard	*Mistelle*	Wine
12 September 2016 42 days *postvéraison*	A	G-AT1	W-AT1.1	6 September 2017 41 days *postvéraison*	C		W-CT1.1
			W-AT1.2				W-CT1.2
			W-AT1.3				W-CT1.3
	B	G-BT1	W-BT1.1				
			W-BT1.2				
			W-BT1.3				
19 September 2016 49 days *postvéraison*	A	G-AT2	W-AT2.1	13 September 2017 48 days *postvéraison*	C	G-CT2	W-CT2.1
			W-AT2.2				W-CT2.2
			W-AT2.3				W-CT2.3
	B	G-BT2	W-BT2.1				
			W-BT2.2				
			W-BT2.3				
26 September 2016 56 days *postvéraison*	A	G-AT3	W-AT3.1				
			W-AT3.2				
			W-AT3.3				
	B	G-BT3	W-BT3.1				
			W-BT3.2				
			W-BT3.3				
3 October 2016 62 days *postvéraison*	A	G-AT4	W-AT4.1	26 September 2017 60 days *postvéraison*	C	G-CT4	W-CT4.1
			W-AT4.2				W-CT4.2
			W-AT4.3				W-CT4.3
	B	G-BT4	W-BT4.1				
			W-BT4.2				
			W-BT4.3				

**Table 2:** Table of correlations between the amino acids in *mistelle* (m) and the difference between the initial amino acid concentration in *mistelle* and final levels in wine (m-w) with fermentative aroma compounds in wine (w). Data are given as Pearson correlation coefficient (R). Significance (*p*-value): **** *p* < 0.001; *** 0.001 < *p* < 0.01; ** 0.01 < *p* < 0.05 and * 0.05 < *p* < 0.1.

	Same Amino Acid (w)	Isobutanol	Isoamyl Alcohol	Methionol	β-Phenyl Ethanol	Isoamyl Acetate	Butyl Acetate	Isobutyl Acetate	Phenylethyl Acetate	Ethyl Isobutyrate	Ethyl 2- Methyl Butyrate	Ethyl Isovalerate	Isobutyric Acid	Isovalerianic Acid	Isobutyr Aldehyde	Isovaler Aldehyde	2-Methyl Butanal	Methional	Phenyl Acetaldehyde
ALA (m)	0.701 **	−0.114	0.119	−0.414	−0.659 **	0.379	0.499	0.283	−0.134	−0.660 **	−0.249	−0.421	−0.121	−0.576 *	0.496	0.517	0.472	−0.009	0.382
ARG (m)	0.468	−0.185	−0.064	−0.290	−0.436	0.413	0.294	0.466	0.152	−0.718 **	−0.472	−0.515	−0.115	−0.340	0.268	0.197	0.309	−0.240	0.015
ASN (m)	0.897 ****	−0.397	−0.292	−0.216	−0.411	0.222	0.143	0.351	0.011	−0.581 *	−0.390	−0.099	−0.229	−0.222	0.373	0.319	0.419	0.118	0.216
ASP (m)	0.385	−0.486	−0.068	0.051	−0.167	0.101	−0.014	0.176	0.008	−0.647 **	−0.576 *	−0.384	−0.404	−0.163	0.038	0.050	0.002	−0.182	−0.027
GABA (m)	−0.632 **	0.657 **	0.291	−0.580 *	−0.450	0.851 ***	0.678 **	0.344	0.215	0.013	0.367	−0.530	0.727 **	−0.425	0.353	−0.222	0.476	−0.523	−0.213
GLU (m)	0.71 3**	0.171	0.049	−0.831 ***	−0.906 ****	0.668 **	0.861 ***	0.462	−0.085	−0.710 **	−0.045	−0.572 *	0.200	−0.842 ***	0.568 *	0.410	0.662 **	−0.189	0.310
GLY (m)	0.735 **	−0.380	0.188	0.219	−0.047	0.030	−0.108	0.095	0.016	−0.573 *	−0.617 *	−0.282	−0.465	−0.072	−0.054	0.169	−0.135	−0.085	0.103
HIS (m)	0.438	−0.115	−0.159	−0.456	−0.501	0.599 *	0.393	0.604 *	0.265	−0.766 ***	−0.436	−0.575 *	0.047	−0.381	0.320	−0.028	0.423	−0.633 **	−0.135
ILE (m)	0.687 **	0.006	−0.069	−0.520	−0.509	0.625 *	0.489	0.529	0.198	−0.807 ***	−0.449	−0.791 ***	0.020	−0.547 *	0.260	−0.154	0.365	−0.789 ***	−0.248
LEU (m)	0.682 **	−0.096	−0.074	−0.473	−0.536 *	0.560 *	0.459	0.499	0.137	−0.832 ***	−0.472	−0.720 **	−0.060	−0.548 *	0.291	−0.006	0.372	−0.719 **	−0.123
LYS (m)	0.780 ***	−0.322	0.185	−0.005	−0.277	0.101	0.124	0.099	−0.130	−0.715 **	−0.575 *	−0.406	−0.561 *	−0.345	0.087	0.329	0.018	−0.092	0.230
MET (m)	0.535	−0.219	−0.054	−0.018	−0.057	0.295	−0.034	0.454	0.340	−0.757 **	−0.779 ***	−0.521	−0.305	−0.071	−0.091	−0.139	−0.051	−0.468	−0.272
ORN (m)	0.218	−0.411	0.168	−0.051	−0.457	−0.229	0.200	−0.358	−0.797 ***	−0.306	−0.038	0.070	−0.516	−0.465	0.469	0.764 **	0.277	0.500	0.765 ***
PHE (m)	0.586 *	−0.062	0.295	−0.050	−0.211	0.187	0.192	0.125	−0.025	−0.661 **	−0.547	−0.696 **	−0.354	−0.376	0.015	0.139	−0.048	−0.420	−0.035
PRO (m)	0.676 **	0.294	0.078	−0.658 **	−0.584 *	0.798 ***	0.665 **	0.567 *	0.258	−0.587 *	−0.160	−0.866 ***	0.359	−0.566 *	0.353	−0.154	0.491	−0.791 ***	−0.280
SER (m)	0.733 **	−0.272	−0.148	−0.046	−0.125	0.334	−0.006	0.513	0.357	−0.707 **	−0.697 **	−0.459	−0.210	−0.050	−0.008	−0.085	0.062	−0.365	−0.243
THR + NH_4_ (m)	0.697 **	−0.307	−0.074	−0.033	−0.324	0.043	0.091	0.113	−0.141	−0.451	−0.368	−0.302	−0.365	−0.244	0.403	0.443	0.344	0.043	0.188
TYR (m)	0.820 ***	−0.290	0.094	−0.107	−0.353	0.189	0.195	0.189	−0.077	−0.712 **	−0.526	−0.490	−0.379	−0.374	0.198	0.294	0.149	−0.165	0.147
VAL (m)	0.691 **	−0.106	−0.123	−0.440	−0.512	0.537	0.413	0.502	0.154	−0.779 ***	−0.465	−0.714 **	−0.061	−0.470	0.343	−0.001	0.422	−0.694 **	−0.150
	Same amino acid (m)	isobutanol	isoamyl alcohol	methionol	β-phenyl ethanol	isoamyl acetate	butyl acetate	isobutyl acetate	phenylethyl acetate	ethyl isobutyrate	ethyl 2- methyl butyrate	ethyl isovalerate	isobutyric acid	isovalerianic acid	isobutyr aldehyde	isovaler aldehyde	2-methyl butanal	methional	phenyl acetaldehyde
ALA (m-w)	0.954 ****	0.042	0.252	−0.481	−0.806 ***	0.369	0.685 **	0.141	−0.274	−0.544	−0.088	−0.514	−0.084	−0.705 **	0.884 ***	0.559 *	0.905 ****	−0.072	0.415
ARG (m-w)	0.999 ****	−0.197	−0.067	−0.260	−0.403	0.403	0.260	0.472	0.173	−0.722 **	−0.501	−0.503	−0.130	−0.308	0.236	0.180	0.650 *	−0.243	−0.001
ASN (m-w)	0.264	0.052	−0.397	−0.671 **	−0.614 *	0.089	0.540	−0.040	−0.428	−0.213	0.232	−0.425	−0.044	−0.664 **	0.693 **	0.238	0.681 **	−0.182	0.050
ASP (m-w)	0.817 ***	−0.551	0.072	0.451	0.188	−0.312	−0.343	−0.293	−0.201	−0.209	−0.396	−0.146	−0.560 *	0.095	−0.097	−0.061	−0.265	−0.061	−0.045
GABA (m-w)	0.944 ****	0.659 *	0.202	−0.447	−0.209	0.551 *	0.483	0.254	0.261	0.187	0.400	−0.406	0.704 **	−0.227	0.154	−0.399	0.275	−0.602 *	−0.362
GLU (m-w)	0.966 ****	0.292	0.122	−0.939 ****	−0.931 ****	0.666 **	0.944 ****	0.363	−0.166	−0.601 *	0.102	−0.640 **	0.269	−0.920 ****	0.723 **	0.371	0.884 ***	−0.263	0.284
GLY (m-w)	−0.339	0.191	0.449	0.352	0.274	−0.511	−0.158	−0.808 ***	−0.506	0.683 **	0.497	0.176	−0.118	0.055	0.040	0.034	−0.198	0.131	0.145
HIS (m-w)	0.890 ****	0.169	−0.117	−0.526	−0.412	0.835 ***	0.427	0.841 ***	0.595 *	−0.710 **	−0.333	−0.559 *	0.418	−0.228	0.204	−0.272	0.401	−0.564 *	−0.344
ILE (m-w)	0.700 **	0.031	−0.003	−0.137	−0.039	0.232	0.125	0.091	0.088	−0.358	−0.309	−0.688 **	−0.099	−0.235	−0.004	−0.526	0.005	−0.778 ***	−0.533
LEU (m-w)	0.736 **	0.081	−0.018	−0.246	−0.145	0.451	0.220	0.349	0.284	−0.465	−0.357	−0.754 **	0.067	−0.234	0.054	−0.501	0.115	−0.810 ***	−0.557 *
LYS (m-w)	−0.058	0.136	0.325	0.633 **	0.749 **	−0.194	−0.573 *	−0.116	0.375	0.216	−0.337	−0.107	−0.130	0.614 *	−0.628 *	−0.584 *	−0.690 **	−0.440	−0.559 *
MET (m-w)	0.784 ***	0.203	0.206	−0.100	0.028	0.666 **	0.091	0.638*	0.625 *	−0.537	−0.555 *	−0.680 **	0.057	−0.050	−0.207	−0.398	−0.154	−0.806 ***	−0.490
ORN (m-w)	0.63 3**	−0.373	0.207	0.403	0.088	−0.792 ***	−0.235	−0.921 ***	−0.879 ***	0.279	0.183	0.212	−0.685 **	−0.119	0.188	0.490	−0.141	0.455	0.496
PHE (m-w)	0.347	0.258	0.366	0.334	0.443	−0.071	−0.223	−0.147	0.203	0.200	−0.085	−0.363	0.035	0.235	−0.393	−0.470	−0.474	−0.521	−0.500
PRO (m-w)	−0.317	0.120	0.364	0.267	0.092	−0.532	−0.048	−0.856 ***	−0.634 **	0.762 ***	0.729 **	0.210	−0.049	−0.028	0.274	0.275	0.015	0.331	0.298
SER (m-w)	0.984 ****	−0.236	−0.133	0.017	−0.037	0.269	−0.073	0.447	0.354	−0.635 **	−0.697 **	−0.496	−0.244	0.001	−0.022	−0.152	0.021	−0.440	−0.331
THR + NH_4_ (m-w)	0.982 ****	−0.248	0.001	−0.025	−0.345	−0.022	0.125	−0.021	−0.262	−0.345	−0.257	−0.331	−0.376	−0.299	0.698 **	0.504	0.660 *	0.052	0.227
TYR (m-w)	0.606 *	−0.081	0.310	0.335	0.208	−0.055	−0.197	−0.089	0.072	−0.169	−0.381	−0.449	−0.284	0.078	−0.114	−0.165	−0.249	−0.401	−0.271
VAL (m-w)	0.871 ****	0.009	−0.043	−0.362	−0.396	0.422	0.370	0.265	0.047	−0.507	−0.271	−0.757 **	−0.023	−0.440	0.388	−0.185	0.595 *	−0.636 **	−0.307

**Table 3:** Table of correlations between the amino acids (Aa) in wine (w) and the most concentrated assimilable amino acids of the must (THR, ARG, ALA, GABA, GLU and SER), and particularly to the ratios of some of them with GABA. Data are given as R^2^ (Linear least-squares regression).

	ARG (m)	THR (m)	ALA (m)	GABA (m)	SER (m)	GLU (m)	THR/GABA (m)	ALA/GABA (m)	GLU/GABA (m)	SER/GABA (m)	(SER + GLU)/GABA (m)	(ALA + SER)/GABA (m)	(ALA + GLU)/GABA (m)	Same Aa/GABA (m)	Same Aa (m)
ASN (w)	0.518 **	0.580 ***	0.448 **	0.192	0.587 ***	0.088	0.624 ***	0.632 ***	0.235	0.678 ***	0.532 **	0.702 ***	0.507 **	0.801 ****	0.787 ****
GABA (w)	0.424 **	0.692 ***	0.542 **	0.401 **	0.345 *	0.113	0.851 ****	0.895 ****	0.364 *	0.590 ***	0.615 ***	0.854 ****	0.739 ****	-	0.401 **
GLU (w)	0.609 ***	0.469 **	0.640 ***	0.059	0.479 **	0.507 **	0.402 **	0.631 ***	0.727 ****	0.451 **	0.826 ****	0.617 ***	0.765 ****	0.727 ***	0.507 **
GLY (w)	0.724 ***	0.500 **	0.518 **	0.072	0.783 ****	0.285	0.434 **	0.547 **	0.452 **	0.717 ***	0.755 ***	0.652 ***	0.578 ***	0.429 **	0.487 **
HIS (w)	0.563 **	0.709 ***	0.613 ***	0.297	0.484 **	0.223	0.812 ****	0.895 ****	0.510 **	0.701 ***	0.798 ****	0.899 ****	0.825 ****	0.757 ****	0.356 *
ILE (w)	0.730 ***	0.463 **	0.768 ****	0.005	0.502 **	0.765 ****	0.268	0.518 **	0.826 ****	0.330 *	0.810 ****	0.489 **	0.726 ***	0.469 **	0.478 **
LEU (w)	0.597 ***	0.554 **	0.772 ****	0.058	0.367 *	0.627 ***	0.479 **	0.757 ****	0.881 ****	0.381 *	0.885 ****	0.671 ***	0.921 ****	0.516 **	0.419 **
LYS (w)	0.571 **	0.595 ***	0.807 ****	0.058	0.320 *	0.614 ***	0.530 **	0.807 ****	0.866 ****	0.363 *	0.862 ****	0.696 ***	0.948 ****	0.566 **	0.619 ***
MET (w)	0.317 *	0.570 **	0.314 *	0.476 **	0.340 *	0.058	0.786 ****	0.667 ***	0.269	0.607 ***	0.534 **	0.702 ***	0.549 **	0.448 **	0.255
PHE (w)	0.624 ***	0.620 ***	0.774 ****	0.075	0.435 **	0.563 **	0.569 **	0.812 ****	0.837 ****	0.480 **	0.925 ****	0.752 ***	0.938 ****	0.415 **	0.352 *
THR (w)	0.602 ***	0.500 **	0.421 **	0.179	0.701 ***	0.164	0.518 **	0.564 **	0.350 *	0.745 ***	0.675 ***	0.674 ***	0.536 **	0.518 **	0.500 **
TYR (w)	0.662 ***	0.603 ***	0.793 ****	0.063	0.460 **	0.604 ***	0.535 **	0.800 ****	0.865 ****	0.483 **	0.947 ****	0.745 ****	0.943 ****	0.643 ***	0.680 ***
VAL (w)	0.660 ***	0.474 **	0.624 ***	0.057	0.567 **	0.482 **	0.402 **	0.615 ***	0.694 ***	0.524 **	0.847 ****	0.634 ***	0.738 ****	0.560 **	0.470 **
ALA (w)	0.599 ***	0.486 **	0.507 **	0.190	0.603 ***	0.232	0.505 **	0.652 ***	0.456 **	0.667 ***	0.735 ***	0.713 ***	0.647 ***	0.652 ***	0.507 **
ASP (w)	0.504 **	0.231	0.470 **	0.011	0.361 *	0.677 ***	0.107	0.273	0.704 ***	0.200	0.626 ***	0.269	0.486 **	0.068	0.143
SER (w)	0.588 ***	0.383 *	0.552 **	0.031	0.543 **	0.418 **	0.322 *	0.528 **	0.578 ***	0.480 **	0.732 ***	0.555 **	0.626 ***	0.480 **	0.543 **
ARG (w)	0.055	0.032	0.200	0.299	0.008	0.447 **	0.003	0.017	0.249	0.069	0.046	0.000	0.091	0.014	0.055
ORN (w)	0.306 *	0.091	0.276	0.052	0.249	0.458 **	0.022	0.112	0.403 **	0.093	0.340 *	0.115	0.240	0.000	0.001
PRO (w)	0.468 **	0.133	0.257	0.041	0.547 **	0.376 *	0.046	0.116	0.340 *	0.290	0.436 **	0.179	0.221	0.561 **	0.469 **

Significance (*p*-value): **** *p* < 0.001; *** 0.001 < *p* < 0.01; ** 0.01 < *p* < 0.05 and * 0.05 < *p*< 0.1.

## Data Availability

Data is contained within the article.

## Supplementary Materials

The following supporting information can be downloaded at: https://www.mdpi.com/article/10.3390/foods11070958/s1, Table S1: Vineyard characterization. List of characteristics which define the selected vineyards; Table S2: Conventional oenological parameters of 33 samples of must expressed as the average and standard deviation (s); Table S3: Conventional oenological parameters, concentration values of volatiles and amino acids found in the set of the 10 *mistelles* (all expressed in micrograms per litre, except the oenological classical parameters); Table S4: Conventional oenological parameters and concentration values of compounds found in the set of the 30 wines (all expressed in micrograms per litre, except the oenological classical parameters). The data were expressed as the average (among replicated measurements) and standard deviation (s); Figure S1: Evolution of some varietal aroma compounds and pH during maturation in *mistelle* samples aged 8 months. 3A, TDN; 3B, γ-nonalactone; 3C, ethyl vanillate; 3D, pH; Table S5: Correlations between amino acid consumed proportions and the quotients 1/GABA, ALA/GABA and GLU/GABA in must. References [51,52,53,54,55] are cited in the supplementary materials.
