# Supplementary material for: Maturation of Moristel in Different Vineyards: Amino Acid and Aroma Composition of Mistelles and Wines with Particular Emphasis in Strecker Aldehydes
Source: Foods. 2022 Mar 25;11(7):958. doi: 10.3390/foods11070958 (PMC8998044; doi:10.3390/foods11070958)
Supplement: Supplementary file 1 [file foods-11-00958-s001.zip › supplementary.pdf]

## Supporting Information for

# Maturation of Moristel in different vineyards. Amino acid and aroma composition of mistelles and wines with particular emphasis in Strecker aldehydes

**Ignacio Arias-Pérez, Ignacio Ontañón, Vicente Ferreira and Ana Escudero***

Laboratory for Aroma Analysis and Enology, Instituto Agroalimentario de Aragón (IA2-Unizar-CITA), Department of Analytical Chemistry, Faculty of Sciences, Universidad de Zaragoza, 50009 Zaragoza, Spain;
iarias@unizar.es (I.A.-P.); ionta@unizar.es (I.O.); vferre@unizar.es (V.F.)
* Correspondence: escudero@unizar.es; Tel.: +34-976-762503; Fax: +34-976-761292

**Table of Contents**


| | Page |
|---|---|
| **Table S1.** Vineyard characterization. List of characteristics which define the selected vineyards. | **S-2** |
| **Table S2.** Conventional oenological parameters of 39 samples of must expressed as the average and standard deviation (s). | **S-3** |
| **Table S3.** Conventional oenological parameters, concentration values of volatiles and amino acids found in the set of the 11 mistelles (all expressed in micrograms per litre, except the oenological classical parameters). | **S-4** |
| **Table S4.** Conventional oenological parameters and concentration values of compounds found in the set of the 39 wines (all expressed in micrograms per litre, except the oenological classical parameters). The data was expressed as the average (among replicated measurements) and standard deviation (s). | **S-9** |
| **Figure S1.** Evolution of some varietal aroma compounds and pH during maturation in mistelle samples aged 8 months. 3A, TDN; 3B, g-nonalactone; 3C, ethyl vanillate; 3D, pH. | **S-12** |
| **Table S5.** Correlations between amino acid consumed proportions and the quotients 1/GABA, ALA/GABA and GLU/GABA in must. | **S-13** |
| **References** | **S-14** |

**Table S1.** Vineyard characterization. List of characteristics which define the selected vineyards.

| | Vineyard | | |
|---|---|---|---|
| | **A** | **B** | **C** |
| **Vineyard conditions** | | | |
| Soil type | Fine sandy loam | Loam | Fine sandy loam |
| Altitude (m) | 463 | 398 | 514 |
| Vineyard age (years) | 38 | 24 | 22 |
| Row orientation | W | SW | SW |
| Vine spacing (mxm) | 3.1x1.2 | 3.3x1.2 | 3.1x1.2 |
| Number of vines / ha | 2366 | 2222 | 2366 |
| **Yield and vegetative growth** | | | |
| Number of bunches in 10 vines | 12.6 | 20.4 | 9.8 |
| Average bunch weight (Kg) | 0.178 | 0.198 | 0.166 |
| Yield (Kg/Ha) | 5306 | 8976 | 3848 |
| Canopy width (m) | 0.5 | 0.6 | 0.4 |
| Canopy height (m) | 0.8 | 1 | 0.8 |
| Canopy solar exposure % | 60 | 20 | 40 |
| Exposed Leaf Area ($m^2$/ vine) | 1.92 | 2.4 | 1.92 |
| Exposed Leaf Area/yield ($m^2$/Kg) | 0.90 | 0.59 | 1.2 |
| **Agricultural practices** | | | |
| Pruning | 4x2 | 6x1 | 4x3 |
| Shoot thinning | Yes | No | Yes |
| Shoot tipping | Yes | Yes | Yes |
| Desuckering | Yes | No | Yes |
| Irrigation | No | No | No |
| Trellising | Double guyot | Double guyot | Double guyot |
| Mulching | No | No | No |
| Leaf plucking | No | No | No |
| Tilling | Yes | Yes | Yes |

**Table S2.** Conventional oenological parameters of 33 samples of must expressed as the average and standard deviation (s).

| Vineyard | A | | | | | | | | B | | | | | | | | C | | | | | |
|---|---|---|---|---|---|---|---|---|---|---|---|---|---|---|---|---|---|---|---|---|---|---|
| Days *Postvéraison* | 42 | | 49 | | 56 | | 62 | | 42 | | 49 | | 56 | | 62 | | 41 | | 48 | | 60 | |
| | **Mean** | **s** | **Mean** | **s** | **Mean** | **s** | **Mean** | **s** | **Mean** | **s** | **Mean** | **s** | **Mean** | **s** | **Mean** | **s** | **Mean** | **s** | **Mean** | **s** | **Mean** | **s** |
| density (g/L) | 1120 | 1.22 | 1121 | 2.04 | 1119 | 3.72 | 1125 | 0.85 | 1108 | 2.03 | 1111 | 1.25 | 1108 | 3.22 | 1117 | 2.06 | 1109 | 3.41 | 1110 | 4.42 | 1119 | 2.08 |
| TSS* (ºBrix) | 25.1 | 0.29 | 25.4 | 0.47 | 25.0 | 0.92 | 26.5 | 0.22 | 22.4 | 0.45 | 23.2 | 0.24 | 22.6 | 0.67 | 24.8 | 0.40 | 22.8 | 0.75 | 23.0 | 1.00 | 25.0 | 0.52 |
| pH | 2.87 | 0.02 | 3.43 | 0.02 | 3.42 | 0.02 | 3.39 | 0.01 | 2.79 | 0.05 | 3.31 | 0.03 | 3.35 | 0.03 | 3.39 | 0.02 | 3.36 | 0.03 | 3.38 | 0.04 | 3.42 | 0.01 |
| total acidity (g tartaric acid/L) | 4.16 | 0.27 | 3.99 | 0.07 | 4.18 | 0.18 | 4.11 | 0.06 | 6.10 | 0.52 | 6.12 | 0.34 | 5.68 | 0.37 | 5.50 | 0.16 | 6.15 | 0.20 | 6.41 | 0.25 | 5.47 | 0.09 |
| YAN** (mg/L) | 328 | 21.4 | 144 | 15.2 | 148 | 10.2 | 186 | 9.1 | 408 | 24.2 | 250 | 21.9 | 233 | 21.1 | 304 | 12.6 | 199 | 19.3 | 234 | 9.1 | 237 | 7.5 |

*TSS: Total soluble solids content

**YAN: yeast assimilable nitrogen

**Table S3.** Conventional oenological parameters, concentration values of volatiles and amino acids found in the set of the 10 *mistelles* (all expressed in micrograms per litre, except the oenological classical parameters).

| Vineyard | A | | | | B | | | | C | |
|---|---|---|---|---|---|---|---|---|---|---|
| Days Postvéraison | 42 | 49 | 56 | 62 | 42 | 49 | 56 | 62 | 48 | 60 |
| Classical parameters | | | | | | | | | | |
| pH | 3.68 | 3.91 | 3.87 | 3.74 | 3.69 | 3.85 | 3.91 | 4.04 | 3.83 | 3.86 |
| TPI | 51 | 57.9 | 57.3 | 57.4 | 48.6 | 57.6 | 57.5 | 59.2 | 39.4 | 46.4 |
| Carbonyl compounds | | | | | | | | | | |
| Fermetative origin | | | | | | | | | | |
| acetoine | 1383 | 2687 | 3768 | 2490 | 289 | 702 | 471 | 548 | 556 | 1101 |
| diacetyl | 15.57 | 25.14 | 46.10 | 26.07 | 9.90 | 14.80 | 2.84 | 15.85 | 12.56 | 17.16 |
| Oxidation-related | | | | | | | | | | |
| acetaldehyde (total) | 10113 | 10482 | 11042 | 10990 | 11591 | 9907 | 10609 | 9808 | 10974 | 10960 |
| acetaldehyde (free) | 1280 | 1309 | 1440 | 1227 | 978 | 1379 | 1353 | 1090 | 1071 | 836 |
| Norisoprenoids | | | | | | | | | | |
| β-damascenone | 8.43 | 5.34 | 7.78 | 8.74 | 11.84 | 11.14 | 9.82 | 11.04 | 7.48 | 7.68 |
| α-ionone | 0.26 | 0.20 | 0.18 | 0.19 | 0.30 | 0.20 | 0.20 | 0.20 | 0.08 | 0.12 |
| β-ionone | 0.75 | 0.75 | 0.71 | 0.66 | 0.75 | 0.76 | 0.74 | 0.75 | 0.68 | 0.69 |
| TDN | 2.11 | 1.29 | 1.24 | 1.77 | 4.01 | 2.84 | 1.99 | 1.61 | 0.95 | 0.94 |
| vitispirane A | 0.073 | 0.022 | 0.033 | 0.052 | 0.075 | 0.060 | 0.035 | 0.026 | 0.006 | 0.006 |
| vitispirane B | 0.048 | 0.016 | 0.022 | 0.034 | 0.053 | 0.041 | 0.025 | 0.020 | 0.006 | 0.006 |
| Riesling acetal | 0.056 | 0.022 | 0.027 | 0.038 | 0.075 | 0.066 | 0.039 | 0.030 | 0.012 | 0.011 |
| Strecker aldehydes | | | | | | | | | | |
| isobutyraldehyde | 5.78 | 11.16 | 14.15 | 19.60 | 10.82 | 22.06 | 10.38 | 25.09 | 15.03 | 18.75 |
| isovaleraldehyde | 7.41 | 14.45 | 16.98 | 18.96 | 15.93 | 27.28 | 5.78 | 32.11 | 19.55 | 28.16 |
| 2-methylbutanal | 2.98 | 6.29 | 8.05 | 11.32 | 4.67 | 10.36 | 5.66 | 11.94 | 6.32 | 10.79 |
| methional | 6.18 | 5.57 | 8.55 | 12.24 | 10.46 | 10.54 | 5.43 | 16.71 | 8.83 | 10.69 |
| phenylacetaldehyde | 3.47 | 13.70 | 4.20 | 6.84 | 2.90 | 11.36 | - | 10.39 | 4.93 | 5.10 |
| Others | | | | | | | | | | |
| (*Z*)-3-hexenal | 0.81 | 0.62 | 0.49 | 0.12 | 0.84 | 0.41 | 0.17 | 0.45 | 0.49 | 0.48 |

| Vineyard | A | | | | B | | | | C | |
|---|---|---|---|---|---|---|---|---|---|---|
| Days *Postvéraison* | 42 | 49 | 56 | 62 | 42 | 49 | 56 | 62 | 48 | 60 |
| Esters | | | | | | | | | | |
| Linear fatty acid derivatives | | | | | | | | | | |
| ethyl propanoate | <LD | <LD | <LD | <LD | <LD | <LD | <LD | <LD | <LD | <LD |
| ethyl butyrate | <LD | <LD | <LD | <LD | <LD | <LD | <LD | <LD | <LD | <LD |
| ethyl hexanoate | 38.1 | 82.7 | 55.2 | 44.6 | 49.7 | 72.4 | 63.7 | 49.1 | 34.4 | 42.7 |
| ethyl octanoate | <LD | <LD | <LD | <LD | <LD | <LD | <LD | <LD | <LD | <LD |
| ethyl decanoate | <LD | 23.3 | <LD | <LD | <LD | 15.9 | <LD | <LD | <LD | <LD |
| Branched acid derivatives | | | | | | | | | | |
| ethyl isobutyrate | 1.98 | 1.69 | 2.18 | 1.94 | 2.20 | 1.85 | 2.08 | 1.88 | 1.77 | 2.20 |
| ethyl 2-methylbutyrate | 0.41 | 0.78 | 0.84 | 0.83 | 0.47 | 0.88 | 0.83 | 0.42 | 0.80 | 0.84 |
| ethyl isovalerate | 0.30 | <LD | <LD | <LD | 0.36 | <LD | <LD | <LD | <LD | <LD |
| ethyl 4-methylpentanoate | <LD | <LD | <LD | <LD | <LD | <LD | <LD | 0.012 | 0.009 | <LD |
| ethyl cyclohexanoate | <LD | 0.044 | <LD | <LD | <LD | <LD | <LD | <LD | 0.034 | <LD |
| Varietal origin | | | | | | | | | | |
| methyl vanillinate | 4.17 | 7.12 | 7.12 | 6.11 | 4.65 | 6.35 | 7.61 | 8.16 | 5.08 | 6.90 |
| ethyl vanillate | 99 | 195 | 265 | 217 | 105 | 228 | 336 | 316 | 122 | 172 |
| ethyl dihidrocinnamate | <LD | <LD | <LD | <LD | <LD | <LD | <LD | <LD | <LD | <LD |
| ethyl cinnamate | 0.29 | 0.29 | 0.50 | 0.57 | 1.09 | 1.66 | 2.05 | 1.95 | 1.09 | 3.20 |
| Fermentative origin | | | | | | | | | | |
| ethyl lactate | 167 | 117 | 125 | 103 | 161 | 178 | 161 | 122 | 119 | 97.4 |
| diethyl succinate | <LD | <LD | <LD | <LD | <LD | <LD | <LD | <LD | <LD | <LD |
| ethyl acetate | 9916 | 10429 | 10363 | 7524 | 6893 | 6737 | 5295 | 3278 | 2982 | 3466 |
| isoamyl acetate | <LD | <LD | <LD | <LD | <LD | <LD | <LD | <LD | <LD | <LD |
| isobutyl acetate | <LD | <LD | <LD | <LD | <LD | <LD | <LD | <LD | <LD | <LD |
| phenylethyl acetate | 3.29 | 2.56 | 3.03 | 3.51 | 4.90 | 5.10 | 4.18 | 4.64 | 2.68 | 2.71 |
| Alcohols | | | | | | | | | | |
| isobutanol | 13.6 | 27.2 | 32.0 | 26.1 | 11.9 | 24.1 | 45.1 | 22.6 | 54.9 | 80.7 |
| 1-butanol | 67.5 | 55.7 | 36.3 | 29.4 | 23.8 | 38.1 | 22.0 | 32.6 | 31.9 | 23.8 |
| isoamyl alcohol | 54.4 | 72.0 | 96.2 | 48.8 | 60.2 | 96.5 | 107.8 | 53.3 | 110.9 | 99.2 |
| methionol | <LD | <LD | <LD | <LD | 17.9 | <LD | <LD | <LD | <LD | 22.2 |
| benzylic alcohol | 67.2 | 117 | 105 | 84.0 | 107 | 154 | 169 | 157 | 213 | 164 |

| Vineyard | A | | | | B | | | | C | |
|---|---|---|---|---|---|---|---|---|---|---|
| Days *Postvéraison* | 42 | 49 | 56 | 62 | 42 | 49 | 56 | 62 | 48 | 60 |
| **Alcohols** | | | | | | | | | | |
| β-phenylethanol | 103 | 118 | 110 | 100 | 138 | 178 | 140 | 171 | 122 | 114 |
| 1-penten-3-ol | 29.1 | 19.8 | 24.6 | 20.0 | 21.7 | 29.2 | 24.7 | 22.8 | 27.9 | 22.5 |
| C6 Alcohols | | | | | | | | | | |
| 1-hexanol | 360 | 561 | 432 | 369 | 387 | 750 | 542 | 648 | 634 | 574 |
| (*Z*)-3-hexenol | 26.3 | 21.2 | 16.3 | 18.9 | 30.4 | 40.0 | 24.9 | 26.3 | 35.3 | 22.5 |
| (*E*)-2-hexenol | <LD | <LD | <LD | <LD | <LD | <LD | <LD | <LD | <LD | <LD |
| (*E*)-3-hexenol | 25.7 | 34.5 | 26.2 | 22.9 | 28.5 | 24.2 | 32.2 | 38.5 | 40.9 | 24.0 |
| Insaturated C8 Alcohols | | | | | | | | | | |
| 1-octen-3-ol | <LD | <LD | <LD | <LD | <LD | <LD | <LD | <LD | <LD | <LD |
| (*E*)-2-octen-1-ol | <LD | <LD | <LD | <LD | <LD | <LD | <LD | <LD | <LD | <LD |
| **Acids** | | | | | | | | | | |
| Linear acids | | | | | | | | | | |
| acetic acid | 55454 | 99997 | 93663 | 45422 | 39513 | 51697 | 38641 | 37585 | 30336 | 37029 |
| butyric acid | <LD | <LD | <LD | <LD | <LD | <LD | <LD | <LD | <LD | <LD |
| hexanoic acid | 77.0 | 217 | 140 | 120 | 87.7 | 208 | 142 | 174 | 110 | 122 |
| octanoic acid | 60.8 | 10.5 | 16.3 | 16.4 | 32.8 | 32.1 | 14.3 | 17.3 | 19.2 | 23.1 |
| decanoic acid | 11.0 | <LD | <LD | <LD | <LD | <LD | <LD | <LD | 23.5 | 13.9 |
| Branched acids | | | | | | | | | | |
| isobutyric acid | 0.063 | 0.093 | 0.111 | 0.086 | 0.034 | 0.070 | 0.082 | 0.065 | 0.097 | 0.102 |
| isovalerianic acid | 22.0 | <LD | <LD | 12.3 | 15.8 | 13.8 | 23.1 | 14.6 | 16.7 | 17.4 |
| **Terpenols** | | | | | | | | | | |
| linalool | 3.59 | 3.71 | 3.52 | 3.75 | 4.84 | 5.19 | 5.20 | 5.97 | 4.26 | 3.52 |
| α-terpineol | 2.51 | 2.11 | 2.29 | 2.90 | 3.46 | 3.08 | 2.16 | 2.06 | 1.93 | 1.80 |
| β-citronelol | <LD | <LD | <LD | <LD | <LD | <LD | <LD | <LD | <LD | <LD |
| geraniol | 3.32 | 13.33 | 4.91 | 5.64 | 4.57 | 5.39 | 6.03 | 8.59 | 5.87 | 6.54 |
| rose oxide | <LD | <LD | <LD | <LD | <LD | <LD | <LD | <LD | <LD | <LD |
| **Lactones** | | | | | | | | | | |
| whiskylactone | 0.32 | 0.32 | 0.30 | 0.16 | 0.32 | 0.31 | 0.24 | 0.18 | 0.28 | 0.32 |
| γ-nonalactone | 5.66 | 3.37 | 2.79 | 2.77 | 5.19 | 3.49 | 2.94 | 1.33 | 1.46 | 0.94 |
| γ-butyrolactone | 936 | 1130 | 1222 | 979 | 1151 | 1568 | 1098 | 1540 | 1475 | 1015 |

| Vineyard | A | | | | B | | | | C | |
|---|---|---|---|---|---|---|---|---|---|---|
| **Days *Postvéraison*** | **42** | **49** | **56** | **62** | **42** | **49** | **56** | **62** | **48** | **60** |
| Volatile phenols | | | | | | | | | | |
| guaiacol | 8.53 | 13.24 | 11.95 | 8.55 | 6.70 | 8.27 | 11.94 | 9.08 | 3.93 | 4.06 |
| *o*-cresol | 0.62 | 0.73 | 0.70 | 0.62 | 1.07 | 1.16 | 1.15 | 1.19 | 0.87 | 0.74 |
| 4-ethylguaiacol | <LD | <LD | <LD | <LD | <LD | <LD | <LD | <LD | <LD | <LD |
| *m*-cresol | 0.27 | 0.22 | 0.29 | 0.23 | 0.37 | 0.36 | 0.29 | 0.27 | 0.22 | 0.21 |
| eugenol | 2.10 | 2.21 | 1.97 | 1.49 | 2.78 | 3.14 | 3.01 | 3.13 | 1.76 | 1.12 |
| 4-ethylphenol | 0.13 | 0.12 | <LD | 0.09 | 0.17 | 0.16 | 0.14 | 0.13 | 0.09 | 0.12 |
| 4-vinylguaiacol | 85.6 | 57.9 | 75.3 | 81.1 | 194 | 217 | 220 | 204 | 54.7 | 56.2 |
| (*E*)-isoeugenol | 1.38 | <LD | <LD | <LD | <LD | <LD | 1.21 | <LD | <LD | <LD |
| 2,6-dimethoxyphenol | 60.7 | 61.8 | 60.4 | 55.1 | 55.8 | 64.1 | 70.0 | 64.9 | 18.6 | 19.4 |
| 4-vinylphenol | 83.7 | 66.0 | 71.3 | 50.9 | 124 | 178 | 138 | 98.9 | 64.9 | 54.7 |
| 4-propylguaiacol | <LD | 0.0021 | 0.0040 | <LD | <LD | <LD | <LD | <LD | <LD | 0.042 |
| 4-alyl-2,6-dimethoxyphenol | <LD | <LD | <LD | 0.76 | 1.37 | <LD | <LD | <LD | <LD | 0.53 |
| vanillin | 83.8 | 106 | 80.1 | 56.2 | 93.2 | 116 | 80.0 | 79.3 | 39.9 | 39.7 |
| acetovanillone | 10.8 | <LD | 10.59 | <LD | 18.2 | <LD | <LD | <LD | <LD | <LD |
| Amino acids | | | | | | | | | | |
| alanine (ALA) | 75590 | 72970 | 76535 | 72149 | 107977 | 130142 | 123442 | 133650 | 132416 | 111788 |
| asparagine (ASN) | 3236 | 1748 | 1652 | 4147 | 12682 | 13814 | 7003 | 13086 | 7695 | 6139 |
| aspartic acid (ASP) | 25376 | 29396 | 28740 | 29429 | 39511 | 59153 | 59983 | 49544 | 31651 | 42118 |
| arginine (ARG) | 63913 | 116483 | 117995 | 144420 | 241301 | 503629 | 429757 | 553273 | 336037 | 266283 |
| cysteine (CYS) | <LD | <LD | <LD | <LD | <LD | <LD | <LD | <LD | <LD | <LD |
| γ-aminobutyric acid (GABA) | 163956 | 187839 | 187316 | 167990 | 139389 | 158668 | 154533 | 191248 | 184088 | 209267 |
| glutamine (GLN) | <LD | <LD | <LD | 6291 | <LD | <LD | <LD | 17692 | <LD | <LD |
| glutamic acid (GLU) | 34683 | 33045 | 36666 | 53914 | 57701 | 55987 | 63875 | 93523 | 96135 | 88015 |
| glycine (GLY) | 4185 | 4867 | 5371 | 3450 | 6833 | 8730 | 10253 | 7662 | 5249 | 5844 |
| histidine (HIS) | 31821 | 32702 | 36640 | 38037 | 38541 | 54743 | 45782 | 60217 | 42386 | 47722 |
| isoleucine (ILE) | 4757 | 6887 | 9337 | 10157 | 7389 | 13018 | 12796 | 15829 | 9786 | 14806 |
| leucine (LEU) | 9554 | 13219 | 16604 | 18940 | 17225 | 28283 | 27544 | 32773 | 21359 | 28442 |
| lysine (LYS) | 5688 | 7698 | 9107 | 7200 | 15309 | 17215 | 23542 | 16283 | 13316 | 13907 |
| methionine (MET) | 3028 | 3642 | 5509 | 4820 | 5153 | 8340 | 8661 | 8937 | 4318 | 5116 |
| ornithine (ORN) | 13379 | 9089 | 9724 | 8368 | 22918 | 21216 | 20520 | 11843 | 21154 | 17601 |

| Vineyard | A | | | | B | | | | C | |
|---|---|---|---|---|---|---|---|---|---|---|
| Days *Postvéraison* | 42 | 49 | 56 | 62 | 42 | 49 | 56 | 62 | 48 | 60 |
| Amino acids | | | | | | | | | | |
| phenylalanine (PHE) | 11022 | 15610 | 17319 | 14952 | 14982 | 20568 | 27191 | 20451 | 18752 | 19320 |
| proline (PRO) | 538462 | 813274 | 812030 | 795274 | 562836 | 895815 | 881742 | 1172368 | 897536 | 1143722 |
| serine (SER) | 28274 | 34659 | 37489 | 37422 | 42998 | 62108 | 57891 | 66441 | 36750 | 39496 |
| threonine-ammonium (THR) | 237135 | 320468 | 278875 | 281212 | 444224 | 582466 | 447672 | 457008 | 440790 | 330583 |
| tyrosine (TYR) | 5815 | 8057 | 8816 | 7942 | 13230 | 18877 | 20880 | 17151 | 13872 | 13635 |
| valine (VAL) | 14013 | 17853 | 20363 | 21934 | 21191 | 32187 | 27846 | 34181 | 24175 | 29101 |

**Table S4.** Conventional oenological parameters and concentration values of compounds found in the set of the 30 wines (all expressed in micrograms per litre, except the oenological classical parameters). The data was expressed as the average (among replicated measurements) and standard deviation (s).

| **Vineyard** | **A** | | | | | | | | **B** | | | | | | | | **C** | | | | | | **Odor thres-hold** [a] |
|---|---|---|---|---|---|---|---|---|---|---|---|---|---|---|---|---|---|---|---|---|---|---|---|
| **Days *Postvéraison*** | **42** | | **49** | | **56** | | **62** | | **42** | | **49** | | **56** | | **62** | | **41** | | **48** | | **60** | | |
| | **Mean** | **s** | **Mean** | **s** | **Mean** | **s** | **Mean** | **s** | **Mean** | **s** | **Mean** | **s** | **Mean** | **s** | **Mean** | **s** | **Mean** | **s** | **Mean** | **s** | **Mean** | **s** | |
| Classical parameters | | | | | | | | | | | | | | | | | | | | | | | |
| pH | 3.34 | 0.02 | 3.35 | 0.03 | 3.37 | 0.02 | 3.37 | 0.03 | 3.36 | 0.02 | 3.30 | 0.05 | 3.40 | 0.03 | 3.43 | - | 3.31 | 0.04 | 3.32 | 0.01 | 3.32 | 0.02 | - |
| volatile acidity (g/L) | 0.637 | 0.040 | 0.570 | 0.070 | 0.515 | 0.049 | 0.727 | 0.193 | 0.527 | 0.021 | 0.490 | 0.000 | 0.550 | 0.020 | 1.085 | - | 0.590 | 0.050 | 0.353 | 0.040 | 0.450 | 0.026 | - |
| total acidity (g tartaric acid/L) | 7.63 | 0.33 | 7.10 | 0.27 | 6.64 | 0.32 | 6.94 | 0.44 | 7.25 | 0.22 | 7.58 | 0.69 | 6.39 | 0.25 | 6.66 | - | 4.49 | 0.31 | 4.51 | 0.19 | 4.56 | 0.13 | - |
| reductive sugar (g/L) | 0.383 | 0.230 | 0.063 | 0.110 | 0.460 | 0.297 | 0.220 | 0.195 | 0.940 | 0.106 | 0.290 | 0.020 | 0.413 | 0.081 | 1.022 | - | 0.923 | 0.259 | 0.667 | 0.501 | 0.993 | 0.202 | - |
| malic acid (g/L) | 0.913 | 0.064 | 0.700 | 0.121 | 0.690 | 0.099 | 0.733 | 0.085 | 0.523 | 0.031 | 0.583 | 0.025 | 0.330 | 0.030 | 0.420 | - | 0.490 | 0.050 | 1.34 | 0.63 | 0.907 | 0.248 | - |
| lactic acid (g/L) | 0.950 | 0.036 | 0.980 | 0.053 | 1.01 | 0.02 | 0.840 | 0.030 | 1.01 | 0.02 | 0.953 | 0.064 | 1.14 | 0.02 | 0.873 | - | 1.22 | 0.06 | 0.907 | 0.219 | 0.910 | 0.046 | - |
| ethanol (%) | 15.7 | 0.1 | 15.4 | 0.16 | 15.69 | 0.86 | 16.78 | 0.35 | 12.6 | 0.2 | 12.65 | 0.27 | 13.13 | 0.46 | 13.97 | - | 12.66 | 0.67 | 13.08 | 0.46 | 14.32 | 0.55 | - |
| TPI | 48.5 | 2.5 | 48.6 | 1.0 | 52.2 | 2.6 | 56.1 | 2.4 | 44.5 | 1.8 | 46.7 | 1.4 | 46.9 | 3.8 | 48.3 | - | 26.4 | 1.9 | 27.1 | 1.4 | 30.0 | 1.3 | - |
| CI | 11.2 | 0.6 | 13.4 | 0.8 | 9.73 | 3.74 | 13.4 | 0.4 | 11.0 | 0.8 | 14.4 | 1.1 | 12.2 | 0.1 | 9.17 | - | 6.20 | 0.42 | 7.21 | 0.78 | 9.14 | 0.53 | - |
| Carbonyl compounds | | | | | | | | | | | | | | | | | | | | | | | |
| Strecker aldehydes | | | | | | | | | | | | | | | | | | | | | | | |
| isobutyraldehyde | 23.3 | 5.5 | 26.1 | 4.2 | 26.2 | 6.9 | 24.1 | 9.2 | 29.0 | 3.6 | 32.8 | 8.0 | 20.6 | 1.5 | 26.8 | - | 38.6 | 4.1 | 36.5 | 4.1 | 34.1 | 3.1 | 6[1] |
| isovaleraldehyde | 18.3 | 2.2 | 20.0 | 9.0 | 15.1 | 5.3 | 8.7 | 1.7 | 38.4 | 4.4 | 24.1 | 5.9 | 26.2 | 0.7 | 13.6 | - | 41.6 | 6.7 | 53.4 | 2.6 | 21.2 | 3.4 | 4.6[1] |
| 2-methylbutanal | 7.53 | 0.70 | 8.82 | 0.73 | 8.26 | 1.43 | 8.52 | 3.39 | 9.47 | 2.15 | 10.19 | 0.21 | 6.27 | 0.27 | 10.12 | - | 11.53 | 3.16 | 11.53 | 1.10 | 11.29 | 1.95 | 16[1] |
| methional | 22.9 | 3.2 | 17.3 | 1.9 | 11.05 | 1.64 | 12.6 | 1.3 | 22.1 | 1.4 | 16.5 | 0.4 | 15.7 | 0.5 | 12.0 | - | 17.7 | 2.9 | 18.8 | 1.2 | 14.6 | 0.6 | 0.5[1] |
| phenylacetaldehyde | 15.1 | 1.9 | 9.7 | 0.9 | 10.2 | 4.0 | 7.0 | 0.4 | 17.0 | 1.8 | 11.3 | 1.1 | 13.1 | 1.1 | 9.3 | - | 16.8 | 4.2 | 18.2 | 3.5 | 14.3 | 2.8 | 1[1] |
| Others aldehydes | | | | | | | | | | | | | | | | | | | | | | | |
| (*Z*)-3-hexenal | 19.8 | 0.6 | 15.7 | 0.6 | 17.2 | 0.7 | 23.6 | 2.3 | 16.9 | 0.2 | 14.9 | 2.2 | 15.8 | 0.9 | 23.7 | - | 17.4 | 0.9 | 17.4 | 0.7 | 19.5 | 0.3 | 0.12[2] |
| Esters | | | | | | | | | | | | | | | | | | | | | | | |
| Branched acid derivatives | | | | | | | | | | | | | | | | | | | | | | | |
| ethyl isobutyrate | 132 | 25 | 147 | 15 | 109 | 6 | 92.2 | 19.7 | 94.4 | 21.4 | 103 | 7 | 70.2 | 12.5 | 59.7 | - | 101 | 6 | 103 | 10 | 85.7 | 4.7 | 15[3] |
| ethyl 2-methylbutyrate | 17.2 | 0.6 | 16.2 | 2.0 | 10.6 | 0.4 | 11.7 | 1.7 | 11.0 | 1.0 | 12.3 | 2.3 | 8.44 | 0.74 | 9.43 | - | 19.0 | 1.0 | 17.0 | 0.2 | 15.9 | 0.5 | 18[3] |
| ethyl isovalerate | 58.1 | 3.8 | 28.2 | 5.3 | 20.5 | 2.1 | 19.2 | 2.6 | 45.1 | 1.9 | 23.6 | 3.8 | 17.4 | 1.5 | 14.2 | - | 20.3 | 0.4 | 16.9 | 0.4 | 15.4 | 1.5 | 3[3] |
| Fermentative origin | | | | | | | | | | | | | | | | | | | | | | | |
| isoamyl acetate | 123 | 14 | 138 | 4 | 171 | 4 | 121 | 50 | 92.9 | 1.3 | 99.3 | 6.3 | 104 | 26 | 285 | - | 232 | 63 | 196 | 72 | 274 | 45 | 30[4] |
| isobutyl acetate | 6.62 | 1.00 | 6.32 | 0.59 | 6.96 | 0.72 | 10.4 | 4.9 | 5.60 | 0.40 | 5.48 | 0.18 | 5.73 | 0.39 | 19.8 | - | 6.22 | 0.47 | 6.23 | 0.76 | 7.79 | 0.05 | 1600[5] |
| phenylethyl acetate | 5.98 | 0.65 | 6.39 | 0.16 | 6.95 | 0.17 | 6.04 | 2.01 | 3.91 | 0.21 | 4.34 | 0.15 | 4.98 | 0.36 | 8.00 | - | 3.05 | 0.26 | 2.68 | 0.25 | 3.31 | 0.38 | 250[4] |

| **Vineyard** | **A** | | | | | | | | **B** | | | | | | | | **C** | | | | | | **Odor threshold** [a] |
|---|---|---|---|---|---|---|---|---|---|---|---|---|---|---|---|---|---|---|---|---|---|---|---|
| **Days *Postvéraison*** | **42** | | **49** | | **56** | | **62** | | **42** | | **49** | | **56** | | **62** | | **41** | | **48** | | **60** | | |
| | **Mean** | **s** | **Mean** | **s** | **Mean** | **s** | **Mean** | **s** | **Mean** | **s** | **Mean** | **s** | **Mean** | **s** | **Mean** | **s** | **Mean** | **s** | **Mean** | **s** | **Mean** | **s** | |
| Alcohols | | | | | | | | | | | | | | | | | | | | | | | |
| isobutanol | 29844 | 1160 | 33245 | 747 | 38116 | 1480 | 31066 | 2176 | 29151 | 1371 | 28198 | 960 | 29192 | 933 | 33961 | - | 31924 | 1195 | 36641 | 4274 | 31887 | 1459 | 40000[4] |
| isoamyl alcohol | 255953 | 10166 | 262100 | 3515 | 281089 | 12159 | 229127 | 9072 | 255297 | 19329 | 245167 | 3437 | 270537 | 9289 | 253260 | - | 255954 | 6558 | 272488 | 12835 | 262139 | 5568 | 30000[4] |
| methionol | 1754 | 137 | 2538 | 1190 | 1967 | 43 | 1036 | 93 | 2066 | 241 | 1859 | 130 | 2316 | 139 | 437 | - | 215 | 4 | 218 | 17 | 184 | 13 | 1000[3] |
| β-phenylethanol | 35143 | 582 | 38796 | 730 | 42320 | 1270 | 33784 | 2781 | 27928 | 1531 | 27190 | 1628 | 35139 | 7627 | 17377 | - | 3440 | 19 | 3521 | 119 | 3188 | 222 | 14000[3] |
| Acids | | | | | | | | | | | | | | | | | | | | | | | |
| Branched acids | | | | | | | | | | | | | | | | | | | | | | | |
| isobutyric acid | 2699 | 216 | 2713 | 256 | 2200 | 5 | 2208 | 300 | 1867 | 63 | 2021 | 207 | 1808 | 55 | 2914 | - | 2692 | 21 | 2491 | 145 | 2585 | 95 | 50[6] |
| isovalerianic acid | 3491 | 135 | 3465 | 231 | 2681 | 99 | 2297 | 159 | 2396 | 39 | 2446 | 115 | 2137 | 53 | 2214 | - | 108 | 5 | 72.3 | 10.5 | 69.9 | 10.2 | 33[3] |
| Amino acids | | | | | | | | | | | | | | | | | | | | | | | |
| alanine (ALA) | 22390 | 162 | 18171 | 3491 | 15110 | 7591 | 21995 | 2226 | 35761 | 3068 | 29575 | 1284 | 34879 | 391 | 41632 | - | 23682 | 2406 | 25380 | 3037 | 19859 | 1528 | - |
| asparagine (ASN) | 5776 | 355 | 4730 | 661 | 4566 | 1042 | 2258 | 1915 | 14454 | 1517 | 12283 | 1019 | 9905 | 904 | 14579 | - | 5175 | 3028 | 5520 | 1572 | 5164 | 1909 | - |
| arginine (ARG) | 11127 | 1860 | 9502 | 940 | 9175 | 3665 | 11785 | 2034 | 6049 | 1401 | 16909 | 4586 | 7742 | 1764 | 12351 | - | 15243 | 4468 | 27819 | 1905 | 24263 | 2175 | - |
| aspartic acid (ASP) | 7774 | 2269 | 7453 | 2786 | 7947 | 5039 | 16562 | 4457 | 17209 | 1483 | 12850 | 473 | 15370 | 2858 | 31872 | - | 16590 | 3716 | 21116 | 8506 | 14453 | 2563 | - |
| cysteine (CYS) | <LD | <LD | <LD | <LD | <LD | <LD | <LD | <LD | <LD | <LD | <LD | <LD | <LD | <LD | <LD | - | <LD | <LD | <LD | <LD | <LD | <LD | - |
| γ-aminobutyric acid (GABA) | 17311 | 1406 | 13553 | 2427 | 10201 | 4745 | 10146 | 476 | 46886 | 4508 | 38177 | 8240 | 37743 | 793 | 28486 | - | 27848 | 2644 | 29412 | 2017 | 17352 | 1776 | - |
| glutamine (GLN) | <LD | <LD | <LD | <LD | <LD | <LD | <LD | <LD | <LD | <LD | <LD | <LD | <LD | <LD | <LD | - | <LD | <LD | <LD | <LD | <LD | <LD | - |
| glutamic acid (GLU) | 12272 | 297 | 9510 | 1879 | 9298 | 5072 | 14473 | 397 | 25544 | 1990 | 18472 | 1617 | 21228 | 1455 | 30735 | - | 18451 | 2155 | 21625 | 3271 | 15398 | 2662 | - |
| glycine (GLY) | 6975 | 362 | 6838 | 2360 | 6508 | 3570 | 10015 | 1158 | 14558 | 1105 | 13857 | 796 | 15333 | 1004 | 21427 | - | 8898 | 1002 | 9856 | 872 | 8519 | 827 | - |
| histidine (HIS) | 4811 | 378 | 4080 | 1429 | 4021 | 2328 | 7200 | 503 | 13471 | 2336 | 13034 | 2108 | 13664 | 554 | 757 | - | 8647 | 1889 | 9350 | 1363 | 8515 | 2020 | - |
| isoleucine (ILE) | 610 | 47 | 338 | 301 | 224 | 364 | 1788 | 412 | 4099 | 895 | 3773 | 815 | 3722 | 484 | 8367 | - | 5502 | 1894 | 5328 | 1462 | 3672 | 940 | - |
| leucine (LEU) | 5222 | 740 | 3584 | 1095 | 2585 | 834 | 6990 | 652 | 15703 | 681 | 11240 | 1206 | 12813 | 1872 | 16119 | - | 14018 | 3533 | 14992 | 3273 | 10156 | 2848 | - |
| lysine (LYS) | 9063 | 1420 | 6925 | 1804 | 5660 | 2500 | 11782 | 1322 | 24273 | 820 | 20418 | 2339 | 21816 | 2554 | 21200 | - | 21869 | 4183 | 23835 | 4010 | 19916 | 5180 | - |
| methionine (MET) | 2358 | 587 | 2147 | 192 | 1908 | 109 | 3201 | 276 | 6052 | 1437 | 5015 | 401 | 3979 | 453 | 3867 | - | 2563 | 746 | 2981 | 1711 | 2772 | 1607 | - |
| ornithine (ORN) | 4125 | 320 | 2961 | 2394 | 2425 | 1812 | 4486 | 655 | 10698 | 9113 | 3096 | 1356 | 3979 | 1318 | 24638 | - | 14268 | 8788 | 8408 | 886 | 8535 | 1655 | - |
| phenylalanine (PHE) | 3972 | 1904 | 4088 | 767 | 4732 | 1772 | 7858 | 636 | 16384 | 868 | 12552 | 2027 | 14713 | 1715 | 14895 | - | 12740 | 2514 | 14072 | 2356 | 11544 | 3317 | - |
| proline (PRO) | 276837 | 55278 | 435702 | 17694 | 659188 | 399625 | 1015638 | 101649 | 821160 | 174531 | 782749 | 333453 | 975427 | 127727 | 2017104 | - | 486432 | 53509 | 581326 | 106862 | 981786 | 176606 | - |
| serine (SER) | 6141 | 1874 | 5850 | 4054 | 4705 | 3228 | 6307 | 264 | 11082 | 643 | 8010 | 273 | 10348 | 2006 | 13669 | - | 9290 | 3312 | 7901 | 2837 | 8281 | 710 | - |
| threonine-ammonium (THR) | 25630 | 3967 | 20493 | 4120 | 16744 | 6945 | 35667 | 6837 | 69435 | 4560 | 58819 | 4709 | 59493 | 4560 | 88683 | - | 48916 | 4462 | 29894 | 5019 | 23221 | 5478 | - |

| Vineyard | A | | | | | | | | B | | | | | | | | C | | | | | | Odor threshold [a] |
|---|---|---|---|---|---|---|---|---|---|---|---|---|---|---|---|---|---|---|---|---|---|---|---|
| Days Postvéraison | 42 | | 49 | | 56 | | 62 | | 42 | | 49 | | 56 | | 62 | | 41 | | 48 | | 60 | | |
| | Mean | s | Mean | s | Mean | s | Mean | s | Mean | s | Mean | s | Mean | s | Mean | s | Mean | s | Mean | s | Mean | s | |
| Amino acids | | | | | | | | | | | | | | | | | | | | | | | |
| tyrosine (TYR) | 3494 | 170 | 2874 | 786 | 2730 | 1388 | 6214 | 544 | 12448 | 688 | 9836 | 1872 | 11579 | 1661 | 12477 | - | 9652 | 2377 | 10974 | 1714 | 9158 | 2279 | - |
| valine (VAL) | 3333 | 315 | 2659 | 981 | 2264 | 1250 | 5146 | 817 | 9251 | 378 | 6624 | 592 | 8489 | 1000 | 12435 | - | 7017 | 1327 | 7350 | 1585 | 5036 | 1109 | - |

*a* Reference in which the odour threshold value has been calculated is given in brackets. [1-6]

**Figure S1.** Evolution of some varietal aroma compounds and pH during maturation in mistelle samples aged 8 months. **A,** TDN; **B**, -nonalactone; **C,** ethyl vanillate; **D**, pH.

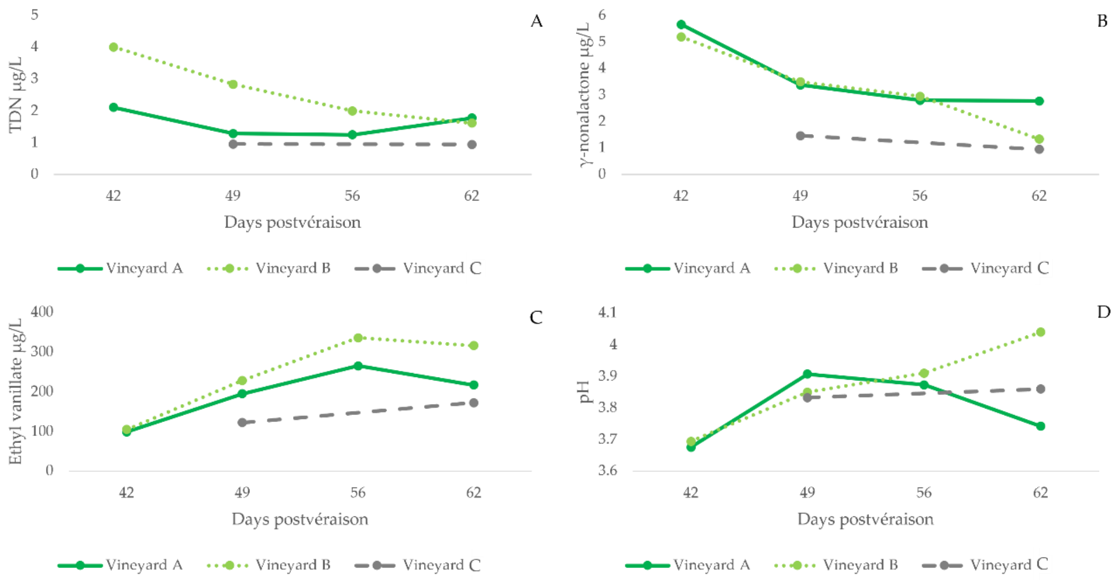

**Table S5.** Correlations between amino acid consumed proportions and the quotients ALA/GABA, 1/GABA and GLU/GABA in must.

| | ALA/GABA | | | 1/GABA | | | GLU/GABA | | |
|---|---|---|---|---|---|---|---|---|---|
| | **$R^2$** | **R** | ***p*** | **$R^2$** | **R** | ***p*** | **$R^2$** | **R** | ***p*** |
| ASN | 0.290 | 0.539 | 0.104 | 0.030 | 0.173 | 0.631 | 0.603 | 0.776 | 0.007 |
| GABA | 0.783 | -0.885 | 0.000 | 0.619 | -0.787 | 0.006 | 0.243 | -0.493 | 0.143 |
| GLU | 0.175 | -0.418 | 0.226 | 0.716 | -0.846 | 0.002 | 0.002 | 0.045 | 0.901 |
| GLY | 0.013 | -0.114 | 0.752 | 0.016 | -0.126 | 0.727 | 0.180 | -0.424 | 0.218 |
| HIS | 0.639 | -0.799 | 0.004 | 0.621 | -0.788 | 0.006 | 0.308 | -0.555 | 0.092 |
| ILE | 0.612 | -0.782 | 0.006 | 0.073 | -0.270 | 0.448 | 0.812 | -0.901 | 0.000 |
| LEU | 0.365 | -0.604 | 0.060 | 0.354 | -0.595 | 0.066 | 0.333 | -0.577 | 0.077 |
| LYS | 0.038 | -0.194 | 0.589 | 0.028 | -0.167 | 0.644 | 0.252 | -0.502 | 0.135 |
| MET | 0.034 | -0.185 | 0.607 | 0.392 | -0.626 | 0.049 | 0.008 | -0.091 | 0.801 |
| PHE | 0.600 | -0.775 | 0.007 | 0.033 | -0.182 | 0.614 | 0.919 | -0.959 | 0.000 |
| THR | 0.222 | -0.472 | 0.165 | 0.201 | -0.448 | 0.190 | 0.189 | -0.434 | 0.206 |
| TYR | 0.197 | -0.444 | 0.194 | 0.128 | -0.357 | 0.307 | 0.505 | -0.711 | 0.019 |
| VAL | 0.468 | -0.684 | 0.026 | 0.324 | -0.569 | 0.082 | 0.465 | -0.682 | 0.027 |
| ALA | 0.025 | -0.157 | 0.664 | 0.401 | -0.633 | 0.046 | 0.000 | -0.012 | 0.973 |
| ASP | 0.019 | -0.138 | 0.703 | 0.031 | 0.177 | 0.624 | 0.414 | -0.644 | 0.041 |
| SER | 0.061 | -0.248 | 0.488 | 0.053 | -0.230 | 0.521 | 0.227 | -0.477 | 0.159 |
| ARG | 0.471 | 0.686 | 0.026 | 0.087 | 0.295 | 0.406 | 0.270 | 0.519 | 0.120 |
| ORN | 0.094 | 0.306 | 0.386 | 0.025 | 0.158 | 0.662 | 0.069 | -0.263 | 0.460 |
| PRO | 0.191 | -0.437 | 0.202 | 0.026 | 0.162 | 0.654 | 0.130 | -0.360 | 0.303 |

## References

1. Culleré, L., Cacho, J., & Ferreira, V. An assessment of the role played by some oxidation-related aldehydes in wine aroma. *J. Agric. Food Chem.* **2007**, *55(3)*, 876-881. doi: 10.1021/jf062432k
2. Sellami, I., Mall, V., & Schieberle, P. Changes in the Key Odorants and Aroma Profiles of Hamlin and Valencia Orange Juices Not from Concentrate (NFC) during Chilled Storage. *J. Agric. Food Chem.* **2018**, *66(28)*, 7428-7440. doi: 10.1021/acs.jafc.8b02257
3. Ferreira, V., López, R., & Cacho, J. F. Quantitative determination of the odorants of young red wines from different grape varieties. *J. Sci. Food Agric.* **2000**, *80(11)*, 1659-1667. doi: 10.1002/1097-0010(20000901)80:11<1659::aid-jsfa693>3.0.co;2-6
4. Guth, H. Quantitation and Sensory Studies of Character Impact Odorants of Different White Wine Varieties. *J. Agric. Food Chem.* **1997**, *45(8)*, 3027-3032. doi: 10.1021/jf970280a
5. Ferreira, V., Ortín, N., Escudero, A., López, R., & Cacho, J. Chemical characterization of the aroma of Grenache rose wines: Aroma extract dilution analysis, quantitative determination, and sensory reconstitution studies. *J. Agric. Food Chem.* **2002**, *50(14)*, 4048-4054. doi: 10.1021/jf0115645
6. Gemert, L. J. N., A. H. (2003). Compilation of odour threshold values in air and water; National Institute for Water Supply: Zeist. Zeist, The Netherlands.
